# Astrocytes in CA1 modulate schema establishment in the hippocampal-cortical neuron network

**DOI:** 10.1186/s12915-022-01445-6

**Published:** 2022-11-10

**Authors:** Shu Liu, Heung Yan Wong, Li Xie, Zafar Iqbal, Zhuogui Lei, Zhongqi Fu, Yuk Yan Lam, Aruna Surendran Ramkrishnan, Ying Li

**Affiliations:** 1 Department of Neuroscience, City University of Hong Kong, Tat Chee Avenue, Kowloon, Hong Kong; 2 Department of Biomedical Sciences, City University of Hong Kong, Tat Chee Avenue, Kowloon, Hong Kong; 3 Centre for Regenerative Medicine and Health, Hong Kong Institute of Science & Innovation, Chinese Academy of Sciences, Hong Kong SAR, Beijing, People’s Republic of China; 4 Centre for Biosystems, Neuroscience, and Nanotechnology, City University of Hong Kong, Tat Chee Avenue, Kowloon, Hong Kong

**Keywords:** Schema, Astrocyte, Hippocampus, Anterior cingulate cortex, Chemogenetics, Calcium imaging

## Abstract

**Background:**

Schema, a concept from cognitive psychology used to explain how new information is integrated with previous experience, is a framework of acquired knowledge within associative network structures as biological correlate, which allows new relevant information to be quickly assimilated by parallel cortical encoding in the hippocampus (HPC) and cortex. Previous work demonstrated that myelin generation in the anterior cingulate cortex (ACC) plays a critical role for dynamic paired association (PA) learning and consolidation, while astrocytes in ACC play a vital role in cognitive decision-making. However, circuit components and mechanism involving HPC-anterior cingulate cortex (ACC) during schema formation remain uncertain. Moreover, the correlation between HPC-ACC circuit and HPC astrocytic activity is unclear.

**Results:**

Utilizing a paired association (PA) behavioral paradigm, we dynamically recorded calcium signals of CA1-ACC projection neurons and ACC neurons during schema formation. Depending on the characteristics of the calcium signals, three distinct stages of schema establishment process were identified. The recruitment of CA1-ACC network was investigated in each stage under CA1 astrocytes Gi pathway chemogenetic activation. Results showed that CA1-ACC projecting neurons excitation gradually decreased along with schema development, while ACC neurons revealed an excitation peak in the middle stage. CA1 astrocytic Gi pathway activation will disrupt memory schema development by reducing CA1-ACC projection neuron recruitment in the initial stage and prevent both CA1-ACC projection neurons and ACC neuron excitation in the middle stage. CA1 astrocytes Gi markedly suppress new PA assimilation into the established memory schema.

**Conclusions:**

These results not only reveal the dynamic feature of CA1-ACC network during schema establishment, but also suggest CA1 astrocyte contribution in different stages of schema establishment.

**Supplementary Information:**

The online version contains supplementary material available at 10.1186/s12915-022-01445-6.

## Background

Memory is a dynamic brain function that tends to be processed after encoding rather than being stored; previous experiences impact the processing of new memories [1]. The framework of pre-existing knowledge, skills or attitudes is called ‘schemas’, a concept from cognitive psychology that explains how new information is integrated with previous experience [2, 3]. Anatomically, schema is defined as the network of neuronal connections which store memory traces of associated information [3]. Current perspectives generally agree that schemas extract features that are common across every individual, that is, once formed, schemas influence memory retrieval strategies and facilitate associative learning [4]. When new experiences are similar to schema predictions, learning may be accelerated as new content can be readily assimilated into existing schemas [5].

Memory consolidation is the process through which memories are stabilized to prevent loss by psychological or neurobiological interventions. Research has put forth two forms of consolidation: synaptic consolidation and systems consolidation. Synaptic consolidation, a process that takes place within minutes to hours of learning, involves protein synthesis and subsequent modifications to the strength of neural connections. Systems consolidation is a long-term reorganization process that takes days to years, during which memory engrams that are initially dependent on the HPC become independent of it and are subsequently represented in the neocortex [5–7]. The neurobiological changes that occur during synaptic consolidation has been studied and described in detail; however, a comprehensive understanding of the mechanisms involved in systems consolidation is still lacking. The formation and updating of memory schema have diverse interactions between HPC and medial prefrontal cortex (mPFC) [5]. It has been proposed that new memories are initially represented within HPC, which are then integrated into a network of existing relevant memories in the neocortex during consolidation [8]. Unlike event-specific memory paradigms, the formation and lasting retention of schematic memory representations in animal models [9–11] has not been well characterized.

An experimental paradigm based on prior knowledge which involves well-learned multiple flavor-location paired association (PA) task establishes a model to explore schematic memory in rodents [10, 11]. These studies have demonstrated that the establishment of schematic memory over several weeks of training is associated with increased myelination in the mPFC [11], a phenomena that has also been observed in a human study [12]. Using immediate early gene (IEG) expression [10] and lidocaine infusion [11], previous studies have suggested that the assimilation of new memories into existing associative memory networks through retrieval-mediated learning requires concurrent encoding in the HPC and mPFC and involves synaptic plasticity [10, 11]. However, the mechanism through which the associative memory networks are formed, and whether the initial network formation depends on retrieval-mediated learning processes backed by dynamic HPC-neocortex interactions is still unknown [13].

As neuroscience research progresses, astrocytes have been found to play a greater role in the central nervous system than merely supporting neurons by providing homeostatic support or encapsulating synapses. Astrocytes are able to manipulate memory processes by both energy metabolism and neurotransmitter-specific effects [14, 15] and exhibit projection-specific effects depending on the input source or the output target of their neighboring neurons [16]. Using chemogenetics as a tool in astrocyte research allows real-time reversible manipulation of astrocytes in tandem with behavioral measurements [17]. Importantly, this reversibility enables the dissection of astrocytes’ effect during different stages of memory in behaving rodents. Recently, Adamsky et al. demonstrated that chemogenetic Gq activation of hippocampal astrocytes enhanced long-term potentiation in CA1 neurons and increased contextual fear memory [18]. Astrocytic Gi activation impaired contextual freezing tested weeks after acquisition associated with a suppression of ACC neuronal activity during retrieval [19]. To determine if astrocytic activity is sufficient to manipulate PA schema memory formation and new memory assimilation, we expressed Designer Receptors Exclusively Activated by Designer Drugs (DREADDs) to activate astrocytic Gi pathway, which has been confirmed to impair memory encoding and recall [19, 20]. In this study, we reveal that activating CA1 astrocyte Gi pathway in every training session disrupted the development of the schema learning and memory retrieval. Neural calcium (Ca^2+^) signaling showed that CA1 astrocytes Gi pathway activation impaired the activities of the learning-induced CA1-ACC projecting neurons in the initial stage of task learning and the middle stage of schema development.

CA1 astrocytes Gi pathway modulation blocked the excitability of both CA1-ACC projecting neurons and ACC neurons, and impaired rapid new associative learning and memory. It is evident that dynamic interactions between both regions are required to develop, represent, and express the features of the schematic memory.

## Results

### CA1 astrocyte Gi activation impairs schema-mediated memory acquisition and expression

**Fig. 1:**
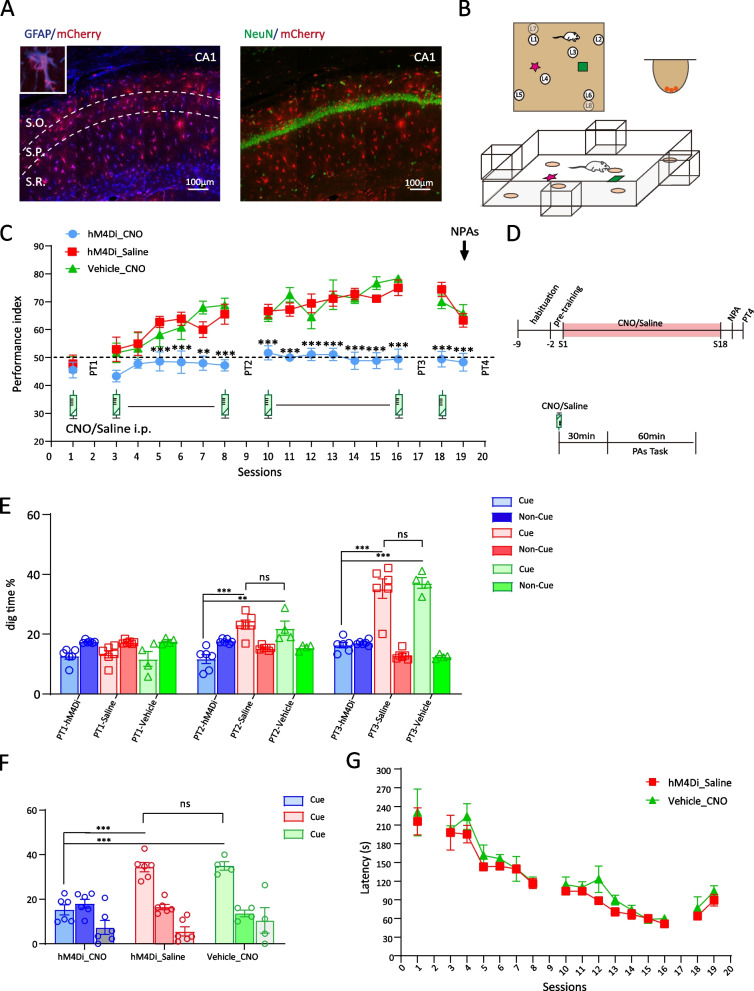
CA1 astrocyte Gi activation impairs schema-mediated memory. **A** Left, virally expressed hM4Di-mCherry in CA1 astrocytic membrane around the soma and in the distal processes. Right, co-localization with the neuronal nuclear marker NeuN (scale bar, 100 µm). **B** Schematic of PA arena, which is composed of six flavor-location paired associations (L1–L6). L7/L8 will replace L1/L6 in new PA training and probe test, 3 pellets were hidden in the bottom of sand well. **C** Performance index during the learning of OPA and NPA in hM4Di_CNO, hM4Di_Saline and Vehicle_CNO groups (hM4Di_CNO and hM4Di_Saline, *n*=6; Vehicle_CNO, *n*=4). CNO or saline was intraperitoneally injected in session 1, sessions 3–8, sessions 10–16, and session 18. **D** Schematic of the experimental protocol, top, timeline of habituation, schema training, NPA introduce, and memory recall. Bottom, timeline of CNO or saline i.p. and tasks training. **E** Nonrewarded cued-recall probe tests (PT1–3) for the acquisition of OPAs across sessions 2, 9, and 17 in hM4Di_CNO (*n*=6), hM4Di_Saline (*n*=6) and Vehicle_CNO (*n*=4) groups. The graph represents the percentage of dig time at the cued location (light color bars) relative to that of the non-cued locations (dark color bars). **F** Memory recall in PT4 for the NPA (light color bar) in hM4Di_CNO (*n*=6), hM4Di_Saline (*n*=6) and Vehicle_CNO groups (*n*=4). **G** The latency (s) rat cost before digging commenced at the correct well in hM4Di_Saline group (*n*=6) and Vehicle_CNO group (*n*=4). Data are presented as the mean ± SEM, **p<0.01, ***p<0.001

To specially modulate CA1 astrocyte Gi pathway activation, we employed an AAV2/9 carrying hM4Di fused to mCherry under the control of the astrocytic GFAP promoter. Vectors of AAV2/9-GFAP-hM4Di-mCherry were injected into the bilateral dorsal HPC/CA1 of rats, resulting in CA1 astrocyte-specific expression which is restricted to the astrocytic outer membranes (Fig. 1A). The expression of vectors has high penetrance (90.78±1.91% of GFAP cells expressed hM4Di), high specificity (92.22±1.05% hM4Di-positive cells were also GFAP-positive) and less leakage of neuron (NeuN showed 1.95±0.23% overlap with hM4Di expression).

To test the effect of astrocytic Gi pathway modulation on overall schema establishment, we prepared two groups of rats (hM4Di_CNO and hM4Di_Saline, *n*=6 each) who received AAV8-GFAP-hM4Di-mCherry bilateral CA1 injection. After 3 weeks of post-surgery recovery and vector expression, 7 days of habituation was conducted and followed by 2 days of pre-training. Rats were trained to learn the schema of six original paired associates (OPAs) over 18 sessions. Each session consisted of six trials. At the beginning of each trial, the rat is given a flavored food pellet as cue in a start box. During each trial, only one sand well out of the six contained the flavored food rewards. Rats were required to dig at one or more sand wells until finding the correct location containing three food pellets of the same flavor as the cue tasted in start box. Three nonrewarded probe tests were conducted to test memory retrieval wherein rats were given a flavor cue and allowed to dig in any of the six sand wells within 120s, however, there would be no food rewards in the correct sand well. The performance index (PI) was calculated by noting the number of incorrect sand wells dug before finding the correct cued location. Memory recall in the nonrewarded probe tests were assessed by calculating the percentage digging time at the correct cued location (Fig. 1B). CNO (10mg/kg, i.p.) in hM4Di_CNO group and same volume of saline in hM4Di_Saline group were administered 30 min before each OPA training session, but not before the probe tests (Fig. 1D). In hM4Di_Saline group, the performance index of OPA training improved steadily across sessions, from chance level (50%) to over 70%, while hM4Di_CNO group lingered at chance level throughout the training sessions. After session 5, performance index in hM4Di_Saline group were significantly higher than hM4Di_CNO group (Fig 1C), indicating that hM4Di_CNO group of rats failed to develop schema-mediated memory of the OPAs.

To assess the memory retrieval of OPAs, 3 nonrewarded probe tests were conducted. The hM4Di_Saline group showed increasing proportion of digging time at the cued location from PT1 to PT3 (PT1 to PT3, 13.32%±1.22 to 35.24%±3.22, F(2,39)= 38.73, *p*<0.001, two-way ANOVA, Tukey’s post hoc, Fig. 1E), while hM4Di_CNO group did not show any improvement in each probe test (F(2,39)= 1.932, *p*=0.158, two-way ANOVA, Tukey’s post hoc, Fig. 1E). In addition, digging time of PT3 in hM4Di_Saline group was noticeably higher than hM4Di_CNO group (hM4Di_Saline vs hM4Di_CNO, 35.24%±3.22 vs 16.35%±0.94, *p*<0.001, two-way ANOVA, Tukey’s post hoc, Fig. 1E), demonstrating that hM4Di_CNO groups did not develop a schema memory of OPAs.

After “schema” is well established, new information will be assimilated rapidly. To verify that rats had successfully developed the schema memory of OPAs, we introduced two new PAs (NPAs), NPA7 and 8 in session 19, followed by NPA probe test in session 20. hM4Di_CNO rats showed poor memory retrieval of NPAs as evidenced by a significantly lower digging time % compared to hM4Di_Saline group (hM4Di_Saline vs hM4Di_CNO, 34.42%±2.10 vs 15.26%±2.26, F(2, 13)=27.854, *p*<0.001, one-way ANOVA, Tukey’s post hoc, Fig. 1F), indicating that hM4Di_CNO group failed to assimilate new information.

CNO is reverse-metabolized to its parent compound clozapine and exerts interoceptive stimulus effects in rats and/or mice [21]. To confirm that the PA learning inhibition was not due to clozapine-like effects, we set another control group (Vehicle_CNO, *n*=4) where rats received the vehicle virus (AAV8-GFAP-mCherry) injection in bilateral CA1. CNO 10mg/kg was i.p. administered 30 min before each training session. Vehicle_CNO group’s performance index or digging time % in PT3 (hM4Di_Saline vs Vehicle_CNO, 35.25%±3.22 vs 37.17%±1.80, *p*=1.000, two-way ANOVA, Tukey’s post hoc, Fig. 1E) showed no difference with hM4Di_Saline group. Vehicle_CNO group was successful in assimilating new PA information; the cued digging time % in PT4 is comparable with that of hM4Di_Saline group (hM4Di_Saline vs Vehicle_CNO, 34.42%±2.10 vs 35.00%±1.91, *p*=0.983, one-way ANOVA, Tukey’s post hoc, Fig. 1F). In addition, both Vehicle_CNO group and hM4Di_Saline group showed no difference in latency which is the time before digging commenced at the correct well during each training session (Fig. 1G), indicating that CNO administration does not impact rat reaction. The above evidences exclude the effect of CNO itself on rats’ behavior.

During consistent Gi pathway activation, hM4Di_CNO group rats neither established schema of OPAs, nor assimilated new information after single session training.

These findings support the hypothesis that CA1 astrocyte activation is necessary for the establishment of memory schema. Next, we investigate the role of CA1 astrocytes in modulating memory transformation between hippocampus and neocortex in the schema development process.

### The feature of CA1-ACC interaction during the development and establishment of memory schema

**Fig. 2:**
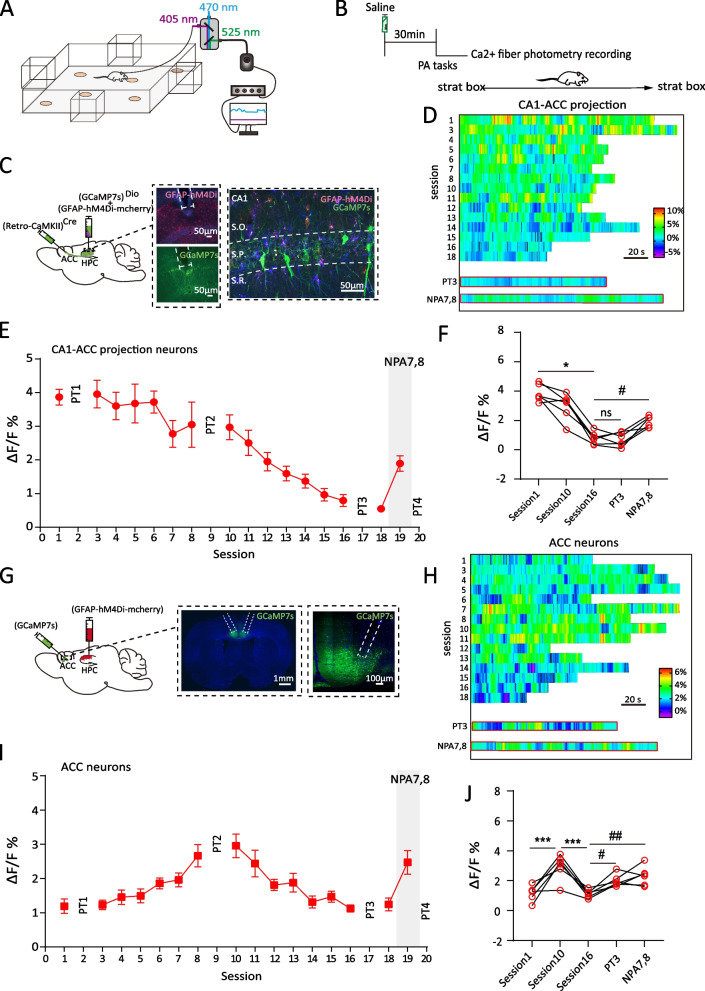
The feature of CA1-ACC interaction during the development and establishment of memory schema. **A** Schematic of the fiber photometry setup and rat moving free in PA arena. **B** Rats received saline i.p. 30 min before each task and fiber photometry recording. **C** Schematic of CA1-ACC projecting neurons experiment: AAV2-retro-CaMKII-Cre was injected into the ACC, and AAV2/9-ef1α-DIO–jGCaMp7s together with AAV8-GFAP-hM4Di–mCherry were injected into the CA1; scale bar, 50 µm. **D** Heatmap of CA1-ACC projecting neurons Ca^2+^ signals transient (Δ*F*/*F*%) for individual rat during session 1 to 18 OPA training, PT3 and NPA training. Column, the typical trial from each session during session 1–18, PT3 and NPA session. Row, time of rat behavior, start from entering PA arena and end at back to the start box. Gradient of heatmap, −5–10% (Δ*F*/*F*%). **E** The average Ca^2+^ signals (Δ*F*/*F*%) of CA1-ACC projecting neurons in session 1–18 OPA training and session 19 NPA training (*n*= 3 rats, trials= 6). **F** Summary plot of averaged Ca^2+^ signals (**E**) in sessions 1, 10, 16, PT3 and NPA. **G** Schematic of ACC neurons experiment: AAV9-Syn-jGCaMp7s was injected into ACC, and AAV8-GFAP-hM4Di–mCherry were injected into the CA1; scale bar, 1mm and 100 µm. **H** The heatmap of ACC neurons Ca^2+^ signals transient (Δ*F*/*F*%) for individual rat during session 1 to 18 OPA training, PT3 and NPA training. Column, the typical trial from each session during sessions 1–18, PT3 and NPA session. Row, time of rat behavior, start from entering PA arena and end at back to the start box. Gradient of heatmap, 0–6% (Δ*F*/*F*%). **I** The average Ca^2+^ signals (Δ*F*/*F*%) of ACC neurons in session 1–18 OPA training and session 19 NPA training (*n*= 3 rats, trials= 6). **J** Summary plot of averaged Ca^2+^ signals (**I**) in sessions 1, 10, 16, PT3 and NPA. Lines connect data collected from the same rat of same trial. Data are presented as the mean ± SEM, *p<0.05. ^#^ *p*<0.05, ^##^*p*<0.01, ns *p*>0.05

Next, we used fiber photometry to assess the Ca^2+^ signal level in CA1 and ACC, correlating the CA1/ACC activity with PA training in freely behaving rats under physiological state (Fig. 2A). Since the process of schema development is a network built between CA1 and ACC, we specifically detected the subpopulation of CA1-ACC projecting cells’ activation. Here, we utilized two new cohorts of rats. The first cohort of rats received bilateral injection of vector AAV2/9-Ef1α-DIO-GCaMP7s and AAV8-GFAP-hM4Di-mCherry into CA1, and simultaneous injection of retro-AAV including the expression of Cre (AAV2-retro-CaMKII-Cre) into ACC. Together, Ca^2+^ indicator GCaMP7s was expressed in CA1-ACC projecting neurons selectively (Fig. 2C). The second cohort of rats were implanted with photometry fibers into bilateral ACC after GCaMP7 infection and AAV8-GFAP-hM4Di-mCherry was injected in CA1 (Fig. 2G). In each session, both cohorts of rats only received saline treatment 30 min before training and recording (Fig. 2B).

Although Ca^2+^ signals (Δ*F*/*F*%) fluctuated during the whole PA task training, it is intriguing that high Ca^2+^ signals/peaks were mostly detected when rats were seeking for the cued location in the open arena or getting food reward pellets. On the contrary, Ca^2+^ signals decreased and then flattened when rats were digging in the sand wells (Additional file 1: Fig S1, 1A). Meanwhile, the probability distribution of averaged *Z*-score of Ca^2+^ signals distinguish these different behaviors (Additional file 1: Fig S1, 1B). Therefore, we only analyzed the average level of Ca^2+^ signal including the rodent behavior of seeking and obtaining food reward pellets, and excluding digging sand wells.

The fiber photometry results showed that Ca^2+^ signals in CA1-ACC projecting neurons were robust when rats were newly exposed into OPAs in session 1 where the Ca^2+^ level (Δ*F*/*F*%) was close to 4% (3.86±0.24%), and then decreased to 2.97±0.37% in session 10. With continuous training of OPA task, Ca^2+^ signals gradually diminished to <1% (0.79±0.17%, Fig. 2E, F) at session 16. In contrast to the varying trend in CA1 Ca^2+^ signals, the Ca^2+^ level in ACC showed a mild increase when first exposed in session 1 (1.19±0.21%), then reached the peak at session 10 (2.95±0.35%). In the subsequent training session, Ca^2+^ level slightly decreased to 1.12±0.11%. Interestingly, when a probe test (PT3) was conducted at the end of training to test the memory recall of OPAs, Ca^2+^ signals in ACC showed an obvious increase compared to session 16 (PT3 vs session 16, 2.01±0.17% vs 1.12±0.11%, *p*=0.042, one-way ANOVA, Tukey’s post hoc, Fig. 2J) while CA1 Ca^2+^ level had no improvement (0.71±0.20%, Fig. 2F), indicating that the ACC, but not CA1, assumes the main role in the memory retrieval of OPA memory retrieval and that information had been transferred and stored into the ACC at the end of schema development. Next, we introduced two NPA7,8 in session 19. Both projection neurons and ACC neurons showed an elevation of Ca^2+^ signals compared to session 16 (session 16 vs NPA; CA1, 0.79±0.17% vs 1.90±0.16%, *p*=0.023; ACC, 2.32±0.26% vs 1.12±0.11%, *p*=0.010; one-way ANOVA, Tukey’s post hoc, Fig. 2E, F, I, J), confirming the parallel encoding in CA1 and ACC during the assimilation of new information. In addition, the possibility of a decrease Ca^2+^ level in CA1 due to photobleaching by prolonged detection was excluded.

These results suggest that memory schema is initially located in CA1 and is gradually transferred to and stored in the ACC. Encoding of new associated information requires parallel activation in both brain areas. We next use three time points (session 1, 10, and 18) to study the process of schema memory development and establishment. CA1 astrocytes will be chemogenetically manipulated in different stages of the process.

### CA1 astrocyte Gi activation impairs the initial stage of memory schema by inhibiting the activation of CA1-ACC projecting neurons (c-Fos expression and calcium level)

**Fig. 3:**
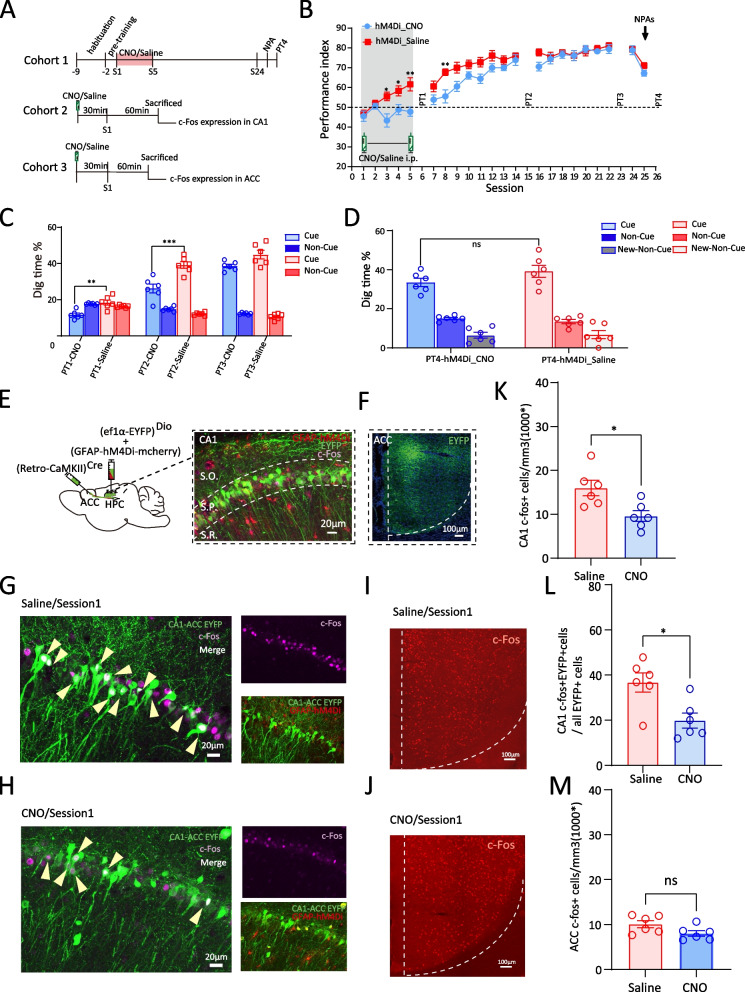
CA1 astrocyte Gi activation impairs the initial schema memory by inhibiting the activation of CA1-ACC projecting neurons (c-Fos expression). **A** Schematic of the experimental protocol. **B** Performance index of Cohort 1 rats during the learning of OPA and NPA in hM4Di_CNO and hM4Di_Saline groups (*n*=6 each). CNO or saline was intraperitoneally injected during session 1 to session 5. **C** Nonrewarded cued-recall probe tests (PT1–3) of Cohort 1 rats for the acquisition of OPAs across sessions 6, 15, and 23 in hM4Di_CNO and hM4Di_Saline groups (*n*=6 each). The graph represents the percentage of dig time at the cued location (light color bars) relative to that of the non-cued locations (dark color bars). **D** Memory recall in PT4 of Cohort 1 rats for the NPA (light color bar) in hM4Di_CNO and hM4Di_Saline groups (*n*=6 each). **E** Schematic of CA1-ACC projecting neurons experiment: AAV2-retro-CaMKII-Cre was injected into the ACC, and AAV2/9-ef1α-DIO-EYFP together with AAV8-GFAP-hM4Di–mCherry were injected into the CA1; scale bar, 20 µm. **F** EYFP-positive axons of CA1 projection neurons in the ACC; scale bar, 100 µm. **G** Representative images of hM4Di in astrocytes (red), EYFP in ACC projecting CA1 neurons (green) and c-Fos (magenta) in the CA1 of saline-injected Cohort 2 rats (**G**) or CNO-injected Cohort 2 rats in session 1 (**H**); white neurons that arrows point to are overlap of EYFP and c-Fos; scale bar, 20 µm. **I** Representative images of c-Fos (red) in ACC of saline-injected Cohort 3 rats in session 1 (**I**) or CNO-injected Cohort 3 rats in session 1 (**J**); scale bar, 100 µm. **K** Comparison of c-Fos expression level in CA1 between hM4Di_CNO and hM4Di_Saline groups of Cohort 2 rats in session 1 (*n*=6 each). **L** Comparison of the percent of CA1 cells projecting into the ACC that express c-Fos between hM4Di_CNO and hM4Di_Saline groups of Cohort 2 rats in session 1 (*n*=6 each). **M** Comparison of c-Fos expression level in ACC between hM4Di_CNO and hM4Di_Saline groups of Cohort 3 rats in session 1 (*n*=6 each). Data are presented as the mean ± SEM, *p<0.05, **p<0.01

We administered CNO or saline i.p. to hM4Di_CNO group and hM4Di_Saline group respectively 30min before training from sessions 1 to 5 (Fig. 3A). The performance index of hM4Di_Saline group during OPA task gradually increased across the 5 sessions, while the performance index of hM4Di_CNO group showed no improvement after session 3 (Fig. 3B). Meanwhile, the retrieval of OPA memory in PT1 also revealed a lower proportion digging time at the cued location in hM4Di_CNO group (PT1, hM4Di_CNO vs hM4Di_Saline, 11.70%±0.92 vs 18.44%±1.40, F(1,30)=8.298, *p*= 0.007, two-way ANOVA, Tukey’s post hoc, Fig. 3C). After withdrawal of CNO i.p., the performance index of hM4Di_CNO group gradually caught up with that of hM4Di_Saline group in session 14, while digging time % in PT2 was still markedly lower (PT2, hM4Di_CNO vs hM4Di_Saline, 26.22%±2.42 vs 39.15%±1.82, F(1,30)=30.526, *p*< 0.001, two-way ANOVA, Tukey’s post hoc, Fig. 3C). Finally, after continuous training for another 7 sessions, CNO rats exhibited a comparable digging time % to the saline group in PT3 at session 23 (Rats’ PA performance recovery was defined as both performance index and probe test digging time % comparable to hM4Di_Saline group). Altogether, these findings indicate that Gi pathway activation of CA1 astrocytes damaged the initial period of schema development, but was recovered after CNO withdrawal.

To examine the activation of the subpopulation of CA1-ACC projecting cells, we tagged these projection neurons with bilateral injection of a retro-AAV which included the expression of the Cre recombinase in excitatory neurons (AAV2-retro-CaMKII-Cre) into the ACC and with a Cre-dependent virus inducing the expression of green fluorescent protein (EYFP) (AAV2/9-Ef1α-DIO-EYFP) into the CA1. AAV8-GFAP-hM4Di–mCherry was simultaneously injected into the CA1 to allow astrocytic manipulation (Fig. 3E). Finally, the three vectors resulted in EYFP expression only in CA1 neurons which projected to ACC, and mCherry expression in CA1 astrocytes (Fig. 3E). Meanwhile, the terminal of projecting fibers from CA1 also showed green fluorescence in ACC (Fig. 3F). This group of rats was then sacrificed 90 min later (Fig. 3A).

We found that in this stage of schema development, CA1 neurons had been dramatically recruited compared to home-cage group (home-cage vs session 1, 6.81×10^3^/mm^3^±0.66 vs 15.97×10^3^/mm^3^±1.74, *p*=0.041, one-way ANOVA, Tukey’s post hoc, Additional file 2: Fig S2, 2B). However, the activation of CA1 neurons was delicate under the manipulation of astrocytes Gi pathway activation. The overall CA1 c-Fos expression in hM4Di_CNO group was significantly downregulated compared to hM4Di_Saline group (hM4Di_CNO vs hM4Di_Saline, 9.58×10^3^/mm^3^±1.21 vs 15.97×10^3^/mm^3^±1.74, *t*(10)=3.012, *p*=0.013, *t*-test, Fig. 3K). More interestingly, the dramatic recruitment during session 1 of OPA task learning was observed in the subpopulation of CA1-ACC projecting cells, with >35% of projection cells expressing c-Fos after OPA task learning, significantly more than home-cage rats (home-cage vs session 1, 6.57%±1.79 vs 36.72%±4.26, *p*<0.001, one-way ANOVA, Tukey’s post hoc, Additional file 2: Fig S2, 2D), and can be downregulated to < 20% by CNO administration (hM4Di_Saline vs hM4Di_CNO, 36.72%±4.26 vs 19.80%±3.32, *t*(10)=3.133, *p*=0.011, *t*-test, Fig. 3L). Identically, results from fiber photometry recording showed that the average Ca^2+^ signals of CA1-ACC projecting neurons were decreased by CNO treatment (hM4Di_Saline vs hM4Di_CNO, 3.49% ±0.28 vs 2.19%±0.32, *t*(32)=3.044, *p*= 0.005, *t*-test, Additional file 3: Fig S3, 3B, 3C and 3E). Furthermore, the probability distribution of Ca^2+^ signals in hM4Di_CNO group showed a left-shift compared to that of hM4Di_Saline group (Additional file 3: Fig S3, 3D), indicating that CNO treatment resulted in decreased Ca^2+^ signal activity in CA1-ACC projecting neurons while learning the OPAs in session 1.

To investigate ACC neuronal activation in the initial stage, we introduced a new cohort of rats which received AAV2/9-GFAP-hM4Di–mCherry infusion in the CA1 and were sacrificed 90min after CNO/Saline i.p. and 60 min after session 1 OPA task training. c-Fos staining and quantification were used to detected ACC neuron activation (Fig. 3A). Compared to the home-cage group, ACC was found to be involved during session 1 OPA task training, as evidenced by increased c-Fos expression (session 1 vs home-cage, 10.08×10^3^/mm^3^±0.79 vs 5.92×10^3^/mm^3^±0.42, *p*=0.018, one-way ANOVA, Tukey’s post hoc, Additional file 2: Fig S2, 2F). However, Gi pathway activation in CA1 astrocytes had no effect on preventing the recruitment of ACC. The c-Fos expression was similar between hM4Di_Saline and hM4Di_CNO groups (hM4Di_Saline vs hM4Di_CNO, 10.08×10^3^/mm^3^±0.79 vs 8.03×10^3^/mm^3^±0.63, *t*(10)= 2.030, *p*= 0.070, *t*-test, Fig. 3M). Consistent with c-Fos expression, no significant effect on ACC neurons’ Ca^2+^ activation was observed in CNO group during learning of OPAs in session 1; the average Ca^2+^ signals did not show a difference between two groups (hM4Di_Saline vs hM4Di_CNO, 1.22%±0.20 vs 1.18%±0.17, *t*(32)=0.1789, *p*=0.859, *t*-test, Additional file 3: Fig S3, 3F, 3G and 3I) or a probability distribution curve shift (Additional file 3: Fig S3, 3H). These data suggest that the recruitment of the subpopulation of CA1-ACC projecting cells is necessary for the initial stage of schema memory learning. To verify whether Gi pathway activation in CA1 astrocytes has an effect on other non-ACC projecting neurons, we tagged additional monosynaptic projections from the CA1 terminating at the nucleus accumbens (NAc) and activated the Gi pathway in CA1 astrocytes. Looking at c-Fos expression in the subpopulation of NAc projecting neurons in session 1, we found that these neurons are only moderately recruited compared to home-cage rats (home-cage vs session 1, 8.40%±0.63 vs 13.12%±1.31, *t*(7)=2.98, *p*=0.021, *t*-test, Additional file 3: Fig S3, 3O). Interestingly, astrocyte Gi pathway activation had no effect on reducing CA1-NAc projecting neurons’ recruitment. (hM4Di_Saline vs hM4Di_CNO, 13.12%±1.31 vs 11.83%±1.53, *t*(7)=0.649, *p*=0.90, *t*-test, Additional file 3: Fig S3, 3K, 3L, 3Q).

These results suggest that astrocyte modulation has distinct effects among different subpopulation neurons in CA1. During schema development, astrocytic Gi pathway activation in CA1 has a specific inhibitory effect on CA1-ACC projecting neurons.

### CA1 astrocyte Gi activation interrupts the middle stage of memory schema formation by impairing CA1-ACC neurons’ interaction (c-Fos expression and calcium level)

**Fig. 4:**
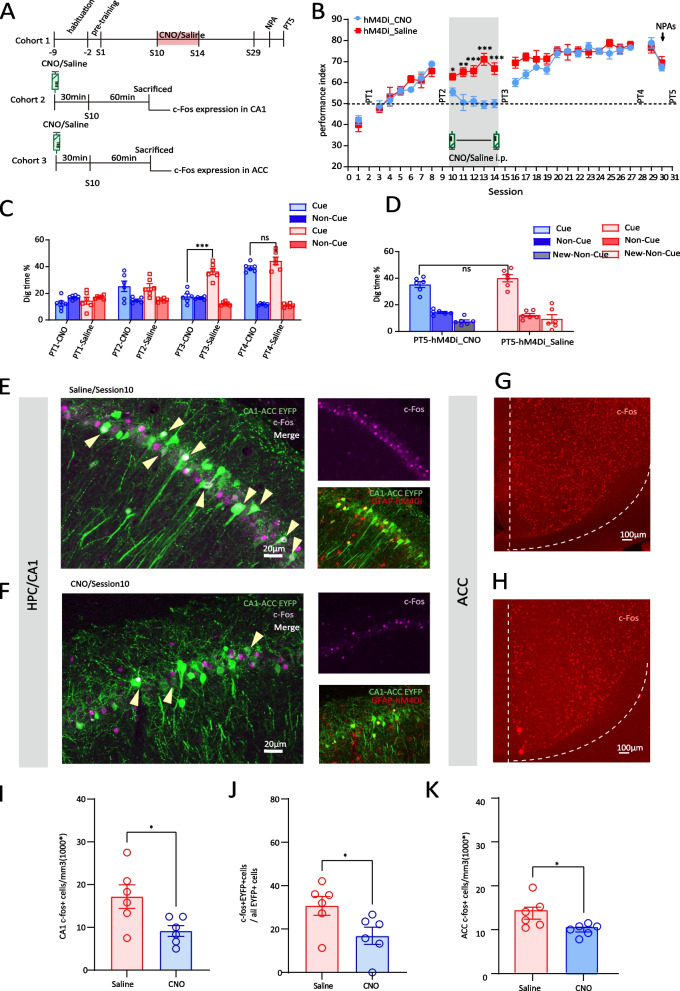
CA1 astrocyte Gi activation interrupts the middle stage of memory schema development by impairing CA1-ACC neurons’ interaction (c-Fos expression). **A** Schematic of the experimental protocol. **B** Performance index of Cohort 1 rats during the learning of OPA and NPA in hM4Di_CNO and hM4Di_Saline groups (*n*=6 each). CNO or saline was intraperitoneally injected during session 10 to session 14. **C** Nonrewarded cued-recall probe tests (PT1–4) of Cohort 1 rats for the acquisition of OPAs across sessions 2, 9, 15, and 28 in hM4Di_CNO and hM4Di_Saline groups (*n*=6 each). The graph represents the percentage of dig time at the cued location (light color bars) relative to that of the non-cued locations (dark color bars). **D** Memory recall in PT5 of Cohort 1 rats for the NPA (light color bar) in hM4Di_CNO and hM4Di_Saline groups (*n*=6 each). **E** Representative images of hM4Di in astrocytes (red), EYFP in ACC projecting CA1 neurons (green) and c-Fos (magenta) in the CA1 of Saline-injected Cohort 2 rats (**E**) or CNO-injected Cohort 2 rats in session 10 (**F**); white neurons that arrows point to are overlap of EYFP and c-Fos; scale bar, 20 µm. **G** Representative images of c-Fos (red) in ACC of saline-injected Cohort 3 rats in session 10 (**G**) or CNO-injected Cohort 1 rats in session 10 (**H**); scale bar, 100 µm. **I** Comparison of c-Fos expression level in CA1 between hM4Di_CNO and hM4Di_Saline groups of Cohort 2 rats in session 10 (*n*=6 each). **J** comparison of the percent of CA1 cells projecting into the ACC that express c-Fos between hM4Di_CNO and hM4Di_Saline groups of Cohort 2 rats in session 10 (*n*=6 each). **K** Comparison of c-Fos expression level in ACC between hM4Di_CNO and hM4Di_Saline groups of Cohort 3 rats (*n*=6 each) in session 10. Data are presented as the mean ± SEM, *p<0.05, **p<0.01, ***p<0.001, ns *p*>0.05

In this study, CNO administration 30min before training from sessions 10 to 14 leads to a gradual decrease in performance index to chance level (Fig. 4B), considerably lower than hM4Di_Saline group. In addition, the digging time % in PT3 of OPA task was also decreased in hM4Di_CNO group (hM4Di_CNO vs hM4Di_Saline, 17.59%±1.96 vs 36.47%±2.43, F(1,40)=27.226, *p*<0.001, two-way ANOVA, Tukey’s post hoc, Fig. 4C). After withdrawal of CNO administration, both the performance index and digging time % in PT could be recovered.

Next, we set up a cohort of CA1-ACC projection-tagged rats which received CNO/Saline i.p. 30 min before session 10 and which were then sacrificed 90 min later (Fig. 4A). Firstly, CA1 was found to be highly involved in the learning of OPAs in session 10 compared to home-cage group, as the evidenced by the overall significantly higher c-Fos expression in CA1 in the home-cage group (home-cage vs session 10, 6.81×10^3^/mm^3^±0.66 vs 17.22×10^3^/mm^3^±2.78, *p*=0.016, one-way ANOVA, Tukey’s post hoc, Additional file 2: Fig S2, 2B). Meanwhile, Gi pathway activation dramatically decreased CA1 neuron recruitment as shown by the significant reduction in c-Fos in hM4Di_CNO group compared to hM4Di_Saline group (hM4Di_Saline vs hM4Di_CNO,17.22×10^3^/mm^3^±2.78 vs 9.17×10^3^/mm^3^±1.26, *t*(10)=2.643, *p*=0.025, *t*-test, Fig. 4E, F, I). Secondly, in the middle stage of schema development, CA1-ACC projecting neurons were evidently recruited, as >30% of CA1-ACC projecting cells expressed c-Fos following task learning, while CNO administration significantly downregulated the proportion of c-Fos in these cells to < 17% (hM4Di_Saline vs hM4Di_CNO, 30.75%±4.38 vs 16.92%±3.95, *t*(10)=2.345, *p*=0.041, *t*-test, Fig. 4E, F, and J). Consistent with the quantification of c-Fos/EYFP overlap, Ca^2+^ levels of CA1-ACC projecting neurons showed a similar trend; the average Ca^2+^ signal recording in session 10 was dramatically decreased after CNO treatment (hM4Di_Saline vs hM4Di_CNO, 2.77%±0.20 vs 1.28%±0.22, *t*(40)=5.017, *p*<0.001, *t*-test, Additional file 4: Fig S4, 4B, 4C and 4E). Furthermore, the probability distribution of Ca^2+^ signals in hM4Di_CNO group was left-shifted compared to that in hM4Di_Saline group (Additional file 4: Fig S4, 4D).

The recruitment of ACC neurons in the learning of OPAs in session 10 is robust, significantly higher than the initial stage (session 10 vs session 1, 14.32×10^3^/mm^3^±1.38 vs 10.07×10^3^/mm^3^±0.79, *p*=0.015, one-way ANOVA, Tukey’s post hoc, Additional file 2: Fig S2, 2F). Unlike the initial stage, the greater involvement of ACC activity in the middle stage of OPA task suggests that ACC may play a critical role in the middle stage of memory schema formation. Moreover, learning-induced elevation of neuron recruitment can be inhibited by CA1 astrocytes Gi pathway activation (c-Fos expression hM4Di_Saline vs hM4Di_CNO, 14.32×10^3^/mm^3^±1.38 vs 10.56×10^3^/mm^3^±0.54, *t*(10)=2.54, *p*=0.029, *t*-test, Fig. 4G, H, K). Identically, in correlation with real-time behavior, Ca^2+^ signal level in ACC was flattened by CNO treatment (hM4Di_Saline vs hM4Di_CNO, 2.54%±0.15 vs 1.33%±0.16, *t*(33)=5.428, *p*<0.001, *t*-test, Additional file 4: Fig S4, 4F, 4G, 4I). In the distribution of probability, the curve of hM4Di_CNO group exhibited a left-shift compared to that of hM4Di_Saline group (Additional file 4: Fig S4, 4H). We show here that chemogenetic manipulation CA1 astrocyte Gi pathway suppressed CA1-ACC projecting neuronal activity and negatively impacts the middle stage task learning by damaging CA1-ACC communication.

### CA1 astrocyte Gi activation has less effects on memory retrieval, but markedly suppresses new PA assimilation into the established memory schema

**Fig. 5:**
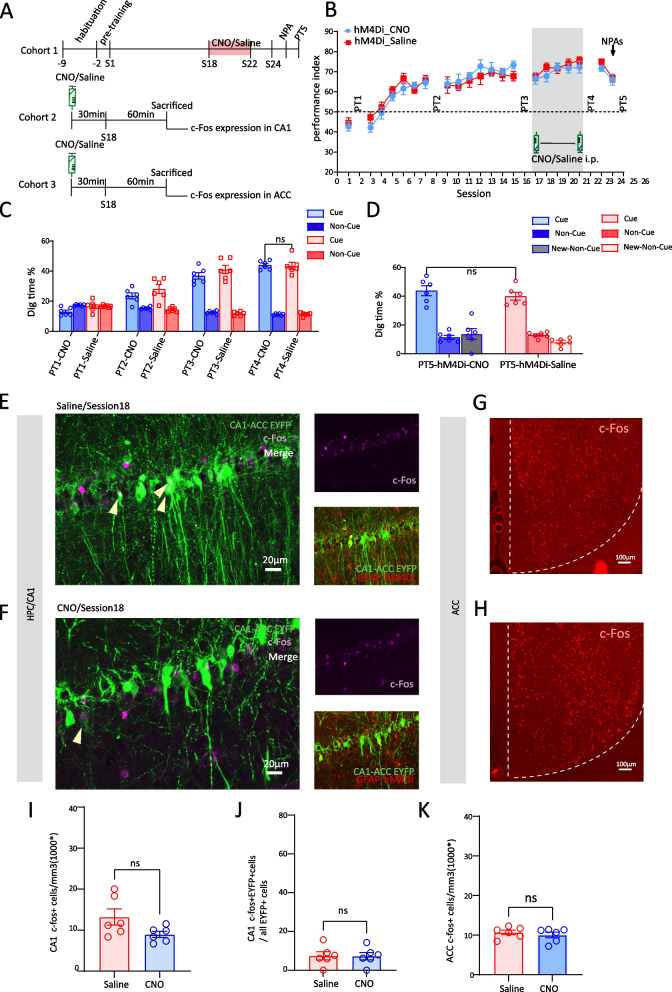
CA1 astrocyte Gi activation has less effects on memory schema in the late stage (c-Fos expression). **A** Schematic of the experimental protocol. **B** Performance index of Cohort 1 rats during the learning of OPA and NPA in hM4Di_CNO and hM4Di_Saline groups (*n*=6 each). CNO or saline was intraperitoneally injected during session 18 to session 22. **C** Nonrewarded cued-recall probe tests (PT1–4) of Cohort 1 rats for the acquisition of OPAs across sessions 2, 9, 17, and 23 in hM4Di_CNO and hM4Di_Saline groups (*n*=6 each). The graph represents the percentage of dig time at the cued location (light color bars) relative to that of the non-cued locations (dark color bars). **D** Memory recall in PT5 of Cohort 1 rats for the NPA (light color bar) in hM4Di_CNO and hM4Di_Saline groups (*n*=6 each). **E** Representative images of hM4Di in astrocytes (red), EYFP in ACC projecting CA1 neurons (green), and c-Fos (magenta) in the CA1 of saline-injected Cohort 2 rats (**E**) or CNO-injected Cohort 2 rats in session 18 (**F**); white neurons that arrows point to are overlap of EYFP and c-Fos; scale bar, 20 µm. **G** Representative images of c-Fos (red) in ACC of saline-injected Cohort 3 rats in session 18 (**G**) or CNO-injected Cohort 3 rats in session 18 (**H**); scale bar, 100 µm. **I** Comparison of c-Fos expression level in CA1 between hM4Di_CNO and hM4Di_Saline groups of Cohort 2 rats in session 18 (*n*=6 each). **J** comparison of the percent of CA1 cells projecting into the ACC that express c-Fos between hM4Di_CNO and hM4Di_Saline groups of Cohort 2 rats in session 18 (*n*=6 each). **K** Comparison of c-Fos expression level in ACC between hM4Di_CNO and hM4Di_Saline groups of Cohort 3 rats (*n*=6 each) in session 18. Data are presented as the mean ± SEM, ns *p*>0.05

After 18 sessions of training with 6 PAs, the food-location associative memory schema was established. To explore the adaptable feature of memory schema and the role of astrocytes in the rapid assimilation of new information into the schema, we administered CNO 30min before sessions 18–22 (Fig. 5A). Unlike the initial and middle stages, late-stage manipulation of CA1 astrocytes had no effect on the performance index of OPA task since the performance index in both groups was ~ 70%, and hM4Di_CNO group showed no difference compared to hM4Di_Saline group (Fig. 5B). The results of PT4 of OPA memory in both groups were maintained in a high level and showed no difference (hM4Di_CNO vs hM4Di_Saline, 44.01%±2.28 vs 43.58±1.18, F(1,40)=0.021, *p*=0.885, two-way ANOVA, Tukey’s post hoc, Fig. 5C). To verify whether astrocytes Gi pathway activation in CA1 will disrupt the recall of schema memory, we set up another cohort of rats who received CNO/saline injection 30 min before sessions 19, 21, 25, and 28, which corresponds to PT4-7 of OPAs (Additional file 5: Fig S5, 5A). The results showed that the memory of OPAs was very robust, and there was no difference in digging time % between PT4-7 (F(3,44)=0.080, *p*=0.971, two-way ANOVA, Tukey’s post hoc, Additional file 5: Fig S5, 5D). Moreover, CA1 astrocytic Gi pathway activation has no effect on memory retrieval of OPAs as no difference was found between hM4Di_Saline group and hM4Di_CNO group (F(1,44)=0.735, *p*=0.396, one-way ANOVA, Tukey’s post hoc, Additional file 5: Fig S5, 5D).

Next, we introduced a cohort of rats which were sacrificed at session 18, 60 min after the task and 90 min after CNO/saline i.p. In hM4Di_Saline group (Fig. 5A), there was an overall increase in c-Fos expression in CA1 compared to home-cage group without statistical significance (home-cage vs session 18, 6.81×10^3^/mm^3^±0.66 vs 13.20×10^3^/mm^3^±1.96, *p*=0.378, one-way ANOVA, Tukey’s post hoc, Additional file 2: Fig S2, 2B), while CNO treatment failed to downregulate c-Fos expression in CA1 (hM4Di_Saline vs hM4Di_CNO, 13.20×10^3^/mm^3^±1.96 vs 8.95×10^3^/mm^3^±0.74, *t*(10)=2.03, *p*=0.07, *t*-test, Fig. 5E, F, and I). Interestingly, the proportion of CA1-ACC projection neuronal recruitment was significantly less than the initial and middle stages and close to the home-cage group with only 7.45% of CA1-ACC projecting cells with c-Fos expression (home-cage vs session 18, 6.57%±1.79 vs 7.45%±2.09, *p*=1.000, one-way ANOVA, Tukey’s post hoc, Additional file 2: Fig S2, 2D). Moreover, c-Fos expression in the subpopulation of CA1-ACC projecting cells had no impact after CNO administration; 7.25% recruitment was observed in hM4Di_CNO group, similar to that of hM4Di_Saline group (hM4Di_Saline vs hM4Di_CNO, 7.45%±2.10 vs 7.25%±1.91, *t*(10)=0.07, *p*=0.945, *t*-test, Fig. 5E, F, and J).

ACC is still involved in session 18 PA task compared to home-cage rats (session 18 vs home-cage, 10.70×10^3^/mm^3^±0.57 vs 5.92×10^3^/mm^3^±0.42, *p*=0.005, one-way ANOVA, Tukey’s post hoc, Additional file 2: Fig S2, 2F). Furthermore, due to low recruitment of CA1-ACC projecting cells, CNO showed limited impact on ACC neuronal activation. The c-Fos expression (hM4Di_Saline vs hM4Di_CNO, 10.70×10^3^/mm^3^±0.57 vs 10.07×10^3^/mm^3^±0.72, *t*(10)=0.6834, *p*=0.510, *t*-test, Fig. 5G, H, K) revealed no difference between hM4Di_Saline and hM4Di_CNO groups.

**Fig. 6:**
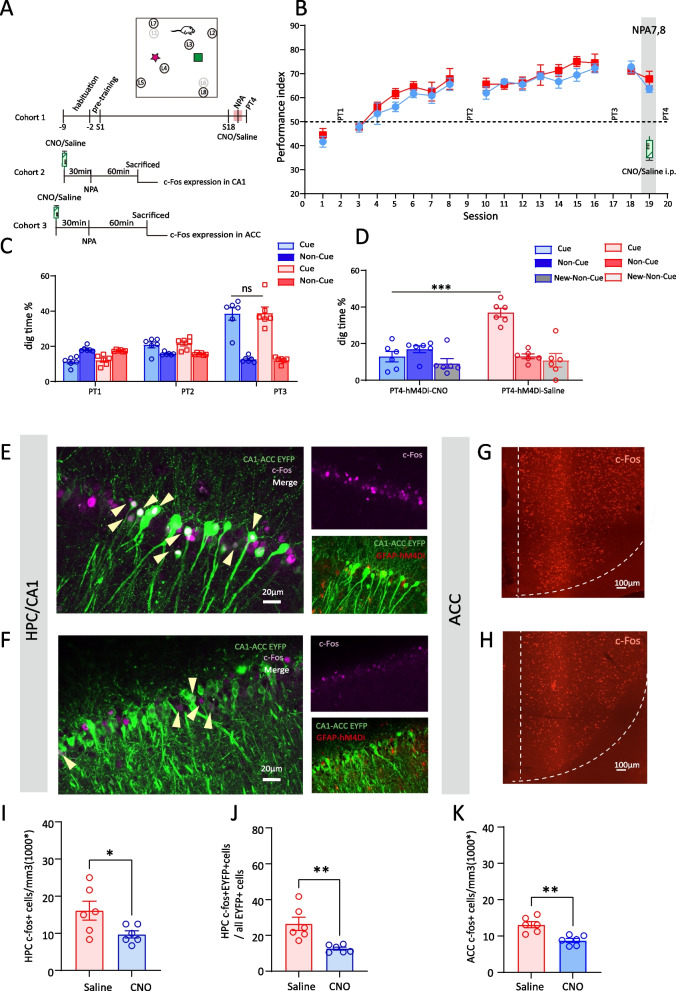
CA1 astrocyte Gi activation markedly suppresses new PA assimilation into memory schema in the late stage (c-Fos expression). **A** Schematic of the experimental protocol. **B** Performance index of Cohort 1 rats during the learning of OPA and NPA in hM4Di_CNO and hM4Di_Saline groups (*n*=6 each). CNO or saline was intraperitoneally injected during NPA training. **C** Nonrewarded cued-recall probe tests (PT1–4) of Cohort 1 rats for the acquisition of OPAs across sessions 2, 9, and 17 in hM4Di_CNO and hM4Di_Saline groups (*n*=6 each). The graph represents the percentage of dig time at the cued location (light color bars) relative to that of the non-cued locations (dark color bars). **D** Memory recall in PT4 of Cohort 1 rats for the NPA (light color bar) in hM4Di_CNO and hM4Di_Saline groups (*n*=6 each). **E** Representative images of hM4Di in astrocytes (red), EYFP in ACC projecting CA1 neurons (green) and c-Fos (magenta) in the CA1 of saline-injected Cohort 2 rats (**E**) or CNO-injected Cohort 2 rats in NPA training (**F**); white neurons that arrows point to are overlap of EYFP and c-Fos; scale bar, 20 µm. **G** Representative images of c-Fos (red) in ACC of saline-injected Cohort 3 rats in NPA (**G**) or CNO-injected Cohort 3 rats in NPA (**H**); scale bar, 100 µm. **I** Comparison of c-Fos expression level in CA1 between hM4Di_CNO and hM4Di_Saline groups of Cohort 2 rats in NPA (*n*=6 each). **J** comparison of the percent of CA1 cells projecting into the ACC that express c-Fos between hM4Di_CNO and hM4Di_Saline groups of Cohort 2 rats in NPA (*n*=6 each). **K** Comparison of c-Fos expression level in ACC between hM4Di_CNO and hM4Di_Saline groups of Cohort 3 rats (*n*=6 each) in NPA. Data are presented as the mean ± SEM, *p<0.05, **p<0.01, ***p<0.001, ns *p*>0.05

Next, we characterize the critical role of CA1 astrocytes in modulating new PA memory encoding after the schema of OPAs had been established. In this cohort of rats, CNO or saline was administered 30min before session 19 during which two new PAs (NPAs) 7 and 8 were introduced, eliminating the former OPAs 1 and 6 (Fig. 6A). We found that performance index of hM4Di_CNO group decreased in session 19, but without statistical significance (hM4Di_Saline vs hM4Di_CNO, 67.79±3.18% vs 63.61±1.52, *t*(10)=1.182, *p*=0.264, multiple *t*-test, Fig. 6B). The probe test of new PAs (PT4) showed that digging time % in hM4Di_CNO group had markedly decreased compared to saline group, suggesting a failure to recall the memory of new PAs (hM4Di_Saline vs hM4Di_CNO, 36.93%±2.41 vs 12.88%±2.84, *t*(10)=0.646, *p*<0.001, *t*-test, Fig. 6D). Another cohort of rats were sacrificed at session 19, 60 min after task training and 90 min after CNO/Saline i.p (Fig. 6A).

The c-Fos expression in CA1-ACC projecting neurons and ACC neurons were examined. We showed significant increases of c-Fos expression in CA1-ACC projecting neurons in NPA acquisition compared to session 18 (NPA vs session 18, 26.47±3.77% vs 7.45±2.10%, *p*=0.005, one-way ANOVA, Tukey’s post hoc, Additional file 2: Fig S2, 2D). Secondly, Gi pathway activation dramatically decreased CA1-ACC projecting neuron recruitment as shown by the significant reduction in c-Fos/EYFP (hM4Di_Saline vs hM4Di_CNO, 26.47±3.77% vs 12.62±0.85%, *t*(10)=3.582, *p*=0.005, *t*-test, Fig. 6E, F, J). ACC also showed sufficient involvement in the acquisition of NPAs as evidenced by a significant increase in c-Fos expression compared to home-cage (NPA vs home-cage, 13.10×103/mm^3^±0.85 vs 5.92×103/mm^3^±0.42, *p*<0.001, one-way ANOVA, Tukey’s post hoc, Additional file 2: Fig S2, 2F). In addition, astrocytic Gi pathway activation in CA1 evidently prevented ACC neurons’ recruitment, shown by the lower level of c-Fos expression (hM4Di_Saline vs hM4Di_CNO, 13.10×10^3^/mm^3^±0.85 vs 8.80×10^3^/mm^3^±0.55, *t*(10)= 4.243, *p*=0.0017, *t*-test, Fig. 6G, H, K).

CA1 astrocyte Gi activation no longer impacts OPA memory retrieval; it appears that when memory schema has been established, the retrieval of OPA memory is independent of CA1. However, when introducing new associated information at this stage, CA1-ACC neural communication will be immediately recruited, and CA1 astrocyte Gi activation exhibit marked suppression on new PA assimilation by disrupting CA1-ACC interaction.

## Discussion

### Prior knowledge as represented by schemas

Memory is continually processed after encoding instead of being stored; past experiences affect how new memories are processed [1]. Moreover, with HPC-driven crosstalk with the neocortex, the presence of schema or a relevant prior associative network can further facilitate memory processing. The HPC plays a critical role in rapid encoding and is necessary for the formation of cohesive memories of distinct events within a context. The mPFC is involved in the configuration of prior knowledge as represented by schemas, which dynamically take part in the encoding of new events and their subsequent retrieval [7]. Unlike event-specific memory paradigms, only a handful of studies have investigated the role of the HPC in schema formation. Recent studies have shown that schema memory establishment over the course of several weeks of training is linked with increased myelination in the mPFC and increases in theta band power, spike-field coherence, and phase locking [7, 11], observations that have also been identified in human studies [12]. In these studies [10, 11, 22], prior knowledge constitutes distinct patterns of flavor-location PAs. In the presence of a well-established memory framework called schema, new information is rapidly integrated, and rats swiftly learn new paired associations within a single trial. In the absence of schema, new associated information will require multiple repeated trainings to be learnt. Importantly, evidence from IEG expression [22] and lidocaine infusion [11] indicate that simultaneous encoding in hippocampus and neocortex may support the integration of rapid new associative memories into existing memory networks, involving synaptic plasticity and myelination of neural circuitry. However, the mechanism for the initial formation of such associative memory networks and whether it depends on retrieval-mediated learning processes supported by hippocampal-neocortex interactions is still unclear [13]. We performed fiber photometry recording of fluorescence from genetic encoded calcium indicators in free-moving behaving rats to examine the real-time neural activities in CA1 and ACC. We found that during the initial learning of OPAs, rats showed greater neural activity input from the hippocampus which contributes to the formation of prefrontal memory networks. Second, we identified a stronger CA1-ACC connectivity in the middle stage of training as the schema memory developed. Third, the CA1 activity progressively diminished once consolidated beyond the hippocampus, and the memories were reliant on the ACC.

Using our model, these findings suggest that the hippocampus promotes the processing of schematic memory through the binding, reconsolidation, and strengthening of connections between neocortical representations, allowing the ACC to retain prior knowledge represented as schemas.

Studies have shown that systems consolidation occurs over a long period of time, during which the role of binding connections may be transferred from HPC to ACC [3, 23–25] and involve the hippocampal–neocortical interactions in the early and middle stages of long-term memory formation [26]. The acquisition of knowledge in a structured manner is supported by the neocortex and is built up over a period of time, resulting in a gradual reduction of hippocampal dependence of the memory trace [3, 8, 10]. This functional change in memory is accompanied by changes in the neural morphology of structures mediating memory [27].

Myelin plasticity is an important feature for learning and various studies have characterized the dynamic nature of myelin [28, 29]. In this context, our published data has shown that the establishment of schematic memory over several weeks of training is accompanied by oligodendrogenesis and adaptive myelination in the ACC network [11], while another human study has detected an increase in neural activation [12]. Myelination is critical for information flow between brain regions that are essential for interactions between different brain regions and for transferring memories. Previous studies have revealed that chronic visceral pain [30] led to suppressed synchronization of theta oscillations between the basolateral amygdala (BLA) and ACC. Moreover, trigeminal neuropathic pain causes hypomyelination in the ACC region [31] and disrupts ACC spike timing and BLA theta oscillation associated with impairment of cognitive function, such as decision-making in rats.

It is noted that human electroencephalogram data has shown that the specific theta oscillatory coherence patterns, e.g. the synchronization and desynchronization, serve an underlying neural network mechanism for instantiation of memory schema in patients with vmPFC damage and controls [32]. Given the fact that paired association (PA) learning and consolidation are associated with increased myelination in the mPFC [9] and that theta oscillations play an important role in the established memory schema in humans, it is conceivable that enhanced myelin strength in the ACC circuitry may contribute to the dynamic nature of CA1-ACC network during PA schema establishment.

Greater activities were found in both CA1 and ACC during new PA learning and memory retrieval. It appears learning in the presence of schema speeds up memory consolidation and new learning is rapidly incorporated into the schemas represented in the ACC. We speculate that the CA1 retrieval-mediated new learning is a two-step process that consists of reactivating existing memories cued by an overlapping new event and a binding method that encodes the association between present events and past experiences. Moreover, in this study, Cre-dependent retrograde tracing suggested that the functional connectivity between these two areas acquired schema expression for learning new information. Importantly, we characterized schema-related ACC activation and new PA-related CA1 reactivation when the animals encoded novel associations that were related to the established memory schema. This is consistent with the notion that the development of schemas involves the ACC and facilitates associative learning mediated by the hippocampus [10, 11, 22].

Using expression of c-Fos, a transcription factor of IEG as a marker of neuronal activity, we observed a higher activation in the hippocampus relative to the ACC during the early period of schema development, and the reverse at the late stage when memory was transformed to established schematic version. In the encoding of the new PA memory, greater increases of c-Fos neurons in both CA1 and ACC were detected. It appears that the onset of new information to an existing schema causes reorganization of related networks. Additional studies are needed to further elucidate the process of consolidating new memories that results in the storage of new and original associations within a common schema. Trans-synaptic anterograde tracers will be used for these further studies.

### Astrocytes are crucial players in schematic learning and memory

Astrocytes play a vital role in the maintenance of synaptic transmission through metabolic network [33]. In recent years, ample evidences have suggested that in order to be fully functional, synapses at neuronal junctions may require signals released from astrocytes [34]. During PA schematic memory tasks, astrocytes may release major gliotransmitters: glutamate, ATP, GABA, and glycine which led to the concept of the tripartite synapse. Their preferential location to the astroglial perisynaptic processes facilitates astrocytic interaction with individual excitatory synapses. Astrocytes can strengthen or diminish both excitatory and inhibitory synaptic transmission, which participate in spike timing-dependent cortical plasticity and working memory [15] and affect behavioral responses involved in cognitive functions [18, 35]. Moreover, astroglial glutamate receptors, particularly NMDA, mGluR3, and mGluR5 types, can activate and modulate extracellular levels of glutamate and activity of neuronal glutamate receptors. This bi-directional glutamatergic communication between astrocytes and neurons contributes to facilitation of schema-mediated memory processing by HPC-ACC neuronal network.

Astrocytes regulate neuronal function and behavior through several intracellular signaling pathways, including Gq-GRCR signaling coupled neurotransmitter receptors and intracellular Ca^2+^-dependent signaling [35]. Using an optogenetic approach, our previous works have revealed that facilitating ACC astrocytic activity increased ACC neuronal spike-theta synchrony and facilitated cognitive performance such as decision-making [36]. It has been demonstrated that Gi pathway activation in astrocytes emulates cellular response to GABAergic stimuli [37, 38] and induces a decrease in the levels of second messenger-cAMP. Meanwhile, CNO administration caused a mild drop in baseline intracellular Ca2+ levels in hM4Di-expressing astrocytes and reduced the total size of Ca^2+^ events in these cells [19]. To determine if astrocytic activity is sufficient to manipulate schematic memory and CA1 neural activity, we applied the DREADD method to inactivate CA1 astrocytes. We showed that prior schema manipulation by CA1 astrocyte Gi activation leads to impaired memory performance associated with suppression of CA1 and ACC neural excitation during multiple PA training, suggesting that it failed to develop schematic memory. Studies have shown that astrocytic glutamate modulates HPC synaptic plasticity by activating postsynaptic NMDA receptors [39, 40] which is necessary in schema PA encoding [22]. It has been reported that astrocytic Gi pathway activation can decrease astrocytic intracellular Ca^2+^ levels and Ca^2+^ events [19], suggesting that the disruption of schema memory by astrocytic Gi pathway modulation may be a result of gliotransmitter release disruption. In the early and middle stages of schema development, CA1 astrocyte Gi pathway activation blocked the dynamic nature of the transformation process by impairing CA1-ACC neural activation strength, suggesting that astrocytic activation is required for reconsolidation in CA1. Interestingly, the effects of astrocytic Gi pathway activation on impaired learning and memory could be restored after discontinuing CNO injection. Since this group of animals may compensate for their inconsistent prior schema, more sessions of training were observed. At the end of PA training, these Gi pathway activation-treated rats were capable of fully establishing memory schema. Moreover, we discovered that rats with astrocyte Gi pathway manipulation in CA1 during new PA task training did not demonstrate new learning, an observation that underscores the critical role of CA1 astrocytes for the rapid assimilation of novel information into existing knowledge frameworks.

The modulation of astrocytes exhibits a certain degree of specificity. Astrocytes exhibit modulation-specific effects depending on the input source or the output target of their neighboring neurons [16]. Kol et al. [19] showed that astrocytes in CA1 can specifically modulate pyramidal cells based on target projection. Recently, we employed double virus strategies combined with chemogenetics to manipulate the BLA-mPFC projecting neurons and showed that chemogenetic activation of BLA astrocytes during fear learning enhances auditory-cued fear memory accompanied by significantly increased c-Fos expression in BLA-PL projecting neurons [41]. In this study, we showed that astrocyte-specific inhibition of PA acquisition is dependent on the projection-specific target. CA1 astrocytes Gi pathway activation decreased ACC projecting cells excitation rather than NAc projecting cells. As a key node of reward circuitry, NAc receives many inputs from CA1, but the subgroup of CA1-NAc projecting cells showed only minor involvement during PA learning than in home-cage. The discrepancy of output target in astrocytic modulation can be explained by the degree of participation of subgroup neurons. In PA learning task, the activation between CA1-ACC circuit has overwhelming superiority, which may demand more energy and gliotransmitter allocation from neighboring astrocytes.

In addition, a single astrocyte can release different gliotransmitters depending on firing frequency and duration of neuronal activity. On acute hippocampus slice, low levels of stimulation of GABAergic interneurons will result in astrocyte glutamate release by activated astrocytic GABAB receptors, while high-frequency interneuron stimulation induces astrocyte release of both glutamate and ATP gliotransmitters. As a consequence, the former had a transient increase in neural synaptic efficacy while the latter resulted in a biphasic transient response, which suggests that astrocytes can decode neuronal input and integrate this information into specific gliotransmitter release. As a relay station, astrocytes greatly enhance the degrees of freedom of the neural circuits and the consequent computational power of the neural systems [42].

### Further questions

It has been suggested that during the transformation process, interactions between the hippocampus and ACC may be facilitated by indirect connections through the entorhinal cortex [43]. The nature of this relaying information and the mechanisms through which it influences the formation of schemas is currently unknown. There remain several other open questions. There is not enough clarity on the nature of ACC memories of new paired associates, and if they are same schematic versions of the original memory. With regard to the time course of hippocampal and prefrontal involvement in memory, longitudinal studies are needed to elucidate whether there is hippocampal activation during retrieval of schema new memory at a week’s delay. Moreover, cross-species research over extended timescales is essential for the differentiation of various models of consolidation.

## Conclusions

A schema is a mental representation of various associated episodes or memories. A dynamic interaction between HPC and ACC and transfer of the binding role from CA1 to the ACC are involved in establishing and expressing the features of long-term schematic memory. During novel experiences, the CA1 and ACC are functionally linked. An established associative memory network in the CA1 and ACC is essential for retrieval-mediated facilitation of new learning. Finally, CA1 astrocytes play a critical role in modulating schematic learning, memory retrieval, and assimilation of new associative memory into existing memory schema.

## Methods

### Animals

All the experimental work was carried out on adult male Sprague Dawley rats (200–300g). They were kept in the cages with 24 h access to food chow and water. The animals were maintained in holding room with a constant room temperature of 25℃, and a 12- to 12-h light and dark cycle. Animal studies were performed in accordance with the guidelines laid down by the Committee on the Use and Care of Animals, Department of Health, Govt. of Hong Kong SAR [Animals (Control of Experiments) Ordinance (Cap. 340), License to Conduct Experiments Ref: (20-19) in DH/HT&A/8/2/5 Pt.1 and (21-47) in DH/HT&A/8/2/5 Pt.5. Approvals for “Ethical Review of Research Experiments involving Animal Subjects” were granted by Animal Research Ethics Sub-Committee, City University of Hong Kong (Ref: A-0170 and A-0513).

### Viruses

The following dilutions and volumes of vectors were used: AAV8-GFAP-mCherry (titer 2.8 × 10^12^, 350nL, bilateral into CA1 per site, Taitool Bioscience); AAV8-GFAP-hM4Di–mCherry (titer 3.5 × 10^12^, 350nL, bilateral into CA1 per site, Taitool Bioscience); AAV2/9-EF1α-DIO-EYFP, (titer 1.3 × 10^12^, 350nL, bilateral into CA1 per site, Taitool Bioscience); AAV2-retro-CaMKIIa-Cre (titer 1.85 × 10^12^, 200nL, bilateral into ACC per site, Taitool Bioscience); AAV2/9-Ef1α-DIO-jGCaMp7s (titer 2.0 × 10^12^, 350nL, bilateral into CA1 per site, Vigene Bioscience); AAV9-Syn-jGCaMp7s (titer 1.8 × 10^12^, 200nL, bilateral into ACC per site, Vigene Bioscience). All viral vectors were aliquoted and stored at −80°C until use.

### Stereotaxic injection

Rats (200–300g) were deeply anesthetized using ketamine (100mg/kg of body weight) and xylazine (8mg/kg). Animals were placed on a stereotaxic frame (RWD instruments). To cover the entire dorsal CA1, four sites per hemisphere were chosen: (1) AP, −3.5mm; ML, ±2.5mm; DV, −2.5mm; from bregma. (2) AP, −3.5mm; ML, ±2.0mm; DV, −2.8mm; from bregma. (3) AP, −4.36mm; ML, ±2.0 mm; DV, −2.6mm; from bregma. (4) AP, −4.36mm; ML, ±3.0 mm; DV, −2.5mm; from bregma. For the ACC the following coordinates were used: (1) AP, −0.36mm; ML, ±0.35 mm; DV, −3.0 mm; from bregma. (2) AP, +0.7 mm; ML, ±0.35 mm; DV, −3.0 mm; from bregma. For the NAc the following coordinates were used: (1) AP, +1.5 mm; ML, ±1.5 mm; DV, −7.0 mm; from bregma. Small volume of virus was carried out using a modified microliter syringe and a 32-gauge metal needle (Hamilton Co., USA). The injection volume and flow rate (100nL/min) were controlled by an injection pump (Hamilton Co., USA). Following each injection, the needle was left in place for an additional 10 min to allow for diffusion of the viral vector away from the needle track and was then slowly withdrawn.

### Fiber optic implantation

In fiber photometry experiments, a fiber optic cannula (OD, 200 µm; 3–5mm length; Inper, Hangzhou, China) was implanted approximately 0.1 mm above the injection site and fixed with dental cement (C&B, Metabond, Parkell, Edgwood, NY) to the skull. The implanted location of CA1 was AP, −3.5mm; ML, ±2.5mm; DV, −2.4mm, and ACC was AP, +0.7 mm; ML,±0.35 mm; DV, −2.9 mm.

### Fiber photometry recording

Fiber photometry allows for real-time excitation and recording of fluorescence from genetic encoded calcium indicators in freely moving rats. Rats were habituated to the fiber patch cord for at least 20 min per day for 2 days before training. Calcium signals were detected using an optical fiber photometry setup (Inper, HangZhou, China). Two excitation wavelengths, 405 and 470 nm, were used in this system, coupled into a 200-µm optical fiber by an objective lens. The laser intensity at the fiber tip was 25–30 µW. GCaMP7s fluorescence was recorded using the same objective, transmitted by the dichroic mirror filtered through a green fluorescence protein bandpass emission filter, and detected by the sensor of a CMOS camera. A labview program was developed to control the CMOS camera, which recorded calcium signals at 100Hz. The acquired raw signals underwent baseline correction of each signal using the adaptive iteratively reweighted penalized least squares (airPLS) algorithm to remove the slope and low-frequency fluctuations in signals [44]. Then motion correction was used to reduce the influence of non-calcium related noise on the results. The 405-nm signal channel was used as the control signal, and the calcium signal data at 470nm were deducted. The Δ*F*/*F*% represented the percentage of fluorescence change. We used 5-s average signals before training when rats stay calm in the start box as *F*, calculated Δ*F*/*F*% as (*F*(*t*)−*F*)/*F* ×100%, and the *t* represented the time.

### CNO administration

Clozapine N-oxide (CNO, Sigma-Aldrich, St Louis, MO, USA) was dissolved in 0.9% NaCl with 10% dimethyl sulfoxide (DMSO). The control also contained 10% DMSO in 0.9% NaCl solution. Then, 10mg/kg CNO was intraperitoneally injected 30 min before behavioral assays. The chosen doses of CNO did not induce any behavioral signs of seizure activity.

### Behavioral test

The protocol of PA behavior is originally developed by Tse et al. [10]. In the PA task, rats are required to learn a map of six flavor-location associations in a large event arena (1.5×1.5 m^2^, Fig. 2A). Sand wells were set up in six different locations, and every location corresponded to one flavor. The entire procedure consists of habituation (−9~−3 session), pre-training (−2 and −1 session), training of original 6 PAs (1–18 sessions), and three nonrewarded probe tests (PT) at sessions 2, 9, and 17 (Fig. 1E).

#### Habituation

During habituation, rats were familiarized with the event arena. One sand well located in the center of arena was opened and 3 normal food pellets were hidden inside it. At the beginning of habituation, the 3 pellets were put at the top of sand well, which were then sequentially moved to the bottom as habituation progressed. At the end of habituation, rats can dig the well and collect food pellets skillfully, then bring them back to the start box and eat them.

#### Training of PAs

The key feature of the protocol was the concurrent training of six flavor-location PA task each day. After two sessions of pre-training (half of flavor-location PAs opened), all PAs were trained. One session consisted by six trials during which PA1 (Marshmallow in L1), PA2 (Chocolate in L2), PA3 (Bacon in L3), PA4 (Strawberry in L4), PA5 (Banana in L5), and PA6 (Beef in L6) were trained. Flavored pellets were brought from Bio-Serv, USA. On any trial, all six sand wells were accessible, but only one sand well contained the food rewards. A trial began with the rats receiving a 0.5g “cue” flavor in the start box. The door of start box was opened after rats has consumed the flavor cue. As the rats come out of start box, they explore the event arena and dig at one or more sand wells until finding the correct location containing three food pellets of the same flavor as the cue consumed in start box. The rats carried back each food reward back to the start box to consume one by one. After a rat returned to the start box with the 3rd pellet, the door was closed. Since rats were trained consecutively, the intertrial interval for an individual rat between successive flavor-location pairings was circa 1 h at initial sessions then close to 30 min towards the culmination of the sessions, a time period in which CNO is confirmed to be working (Fig. 1F). In addition, to ensure that rats located the correct sand well by utilizing their memory of the flavor-location associations and not by the scent of the food pellets, pellets were ground up and mix in the sand (25 g/2.5 kg sand) before start of each session. The acquisition performance of PAs was measured by performance index (PI), that is the number of incorrect sand wells rats dug in before choosing the correct sand well. It was calculated as (100% − number of errors × 20%).

#### New PA acquisition

Once rats had acquired schema map of the six original PAs, two new PAs will be introduced. PA7 (Very berry in L7) replaced PA1, and PA8 (Pino colada in L8) replaced PA6. New PAs are only trained within a single session and followed a probe test 24h later by using one of the new PAs as the flavor cue.

#### Nonrewarded probe test

To assess cued-recall memory, nonrewarded probe test (PT) was scheduled. During this test, all six sand wells were open as usual for the rats to dig, but none contained any food reward. After the start box was opened, rats were allowed explore in arena for a total of 120s. If rat did not dig any well during 120s, a further 60 s was given before ending the test. Memory retrieval performance was measured by calculating the percentage digging time (dig time %) at the cued location and the percentage digging time at the non-cued locations, since rats with good memory would dig the correct cued sand well for a longer time. The rats were provided with 3 pellets of the cued flavor in the correct sand well after the PT to limit the extinction of memory.

### Immunohistochemistry and histology

Rats were transcardially perfused with cold PBS followed by 4% paraformaldehyde (PFA) in PBS when sacrificed. The brains were extracted, postfixed overnight in 4% PFA at 4℃, then cryoprotected in 30% sucrose in PBS for 5 days. Brains were sectioned to a thickness of 35 µm using a sliding freezing microtome (Leica SM2010R) and preserved in a cryoprotectant 30% ethylene glycol, in PBS. Free-floating sections were washed in PBS, incubated for 1 h in blocking solution (10% normal goat serum (NGS) and 0.3% Triton X-100 in PBS), and incubated overnight at 4℃ with primary antibodies (mouse anti-GFAP, Millipore, 1:500; rabbit anti-NeuN, Cell Signaling Technology, 1:500; rabbit anti c-fos, Synaptic Systems, 1:500) in 0.1% Triton and 3% NGS in PBS. Sections were then washed with PBS and incubated for 2 h at room temperature with secondary antibodies (Goat anti-mouse, Alexa Fluor 405, 1:500; goat anti-rabbit, Alexa Fluor 488, 1:500; goat anti-rabbit, Alexa Fluor 594, 1:500; donkey anti-rabbit, Alexa Fluor 647, 1:600) in 3% NGS in PBS. Finally, sections were washed in PBS, incubated with DAPI (1mg/ml), mounted on slides and sealed with mounting medium (Flouromount-G, eBioscience, San-Diego, CA, USA). Then, the stained brain slices were scanned by a confocal microscope (LSM 880; Carl Zeiss, Oberkochen, Germany), using ×10 or ×20 air objectives and ×63 oil-immersion objectives. A total of 3–5 sections were scanned per rat, and 6 rats were studied per group. Confocal images obtained as z-stacks of 10 images collected at 1 µm steps.

### Quantification and statistical analysis

All data were presented as mean ± SEM, and comparisons were conducted with two-tail Student’s *t* test, factorial ANOVA followed by post hoc tests, or Wilcoxon-signed rank test where appropriate, using SPSS v.19.0 (IBM SPSS, Chicago, IL, USA) or Prism v.9.0 (GraphPad Software, La Jolla, CA, USA). To compare the distribution of Ca^2+^ signal level between hM4Di_CNO and hM4Di_Saline group, Ca^2+^ signals normalized to Δ*F*/*F*% for each rat first. Then, the minimum and maximum values of the Δ*F*/*F*% for all rats were determined and used as the full range for binning. The probability distributions of Δ*F*/*F*% from hM4Di_CNO and hM4Di_Saline group were constructed separately. For each area, the mean and standard deviations (SD) of the corresponding Δ*F*/*F*% proportions were calculated across all rats. To compare the probability distribution of Ca2+ signals level between seeking + get reward behaviors and digging behavior in one trial of each rat, we normalized Δ*F*/*F*% to *Z*-score, next steps are the same as above. The probability of distribution was conducted by Matlab (MathWorks, Natick, MA, USA).

## Supplementary Information

**Additional file 1:** **Fig S1.** Calcium signals distribution during the trials of PA training. (A) Representative Ca2+ transient during one trial in PA training; Ca2+ signals’ peak present to seeking and get rewarding behaviors, and be flatten at digging behavior. (B) Probability distribution of averaged Z-score of Ca2+ signals in seeking + get rewarding behaviors and digging behavior for normal rats in session 1 (*n*=4, trials=16).

**Additional file 2:** **Fig S2.** Comparison of c-Fos expression between home-cage and other sessions. (A) Representative images of hM4Di in astrocytes (red), EYFP in ACC projecting CA1 neurons (green) and c-Fos (magenta) in the CA1 of Saline-injected home-cage rats (A) or CNO-injected home-cage rats (C); white neurons that arrows point to are overlap of EYFP and c-Fos; scale bar, 20 µm. (B) Comparison of c-Fos expression level in CA1 between home-cage and sessions 1, 10, 18, NPA. (D) Comparison of the percent of CA1 cells projecting into the ACC that express c-Fos between home-cage and sessions 1, 10, 18, NPA. (E, F) Comparison of c-Fos expression level in ACC between home-cage and sessions 1, 10, 18, NPA. (G) The scheme of microdialysis probe insert and perfusion in CA1 and ACC. Data are presented as the mean ± SEM, *p<0.05, **p<0.01, ***p<0.001; #*p*<0.05, ##*p*<0.01.

**Additional file 3:** **Fig S3.** CA1 astrocyte Gi activation impairs initial schema memory by inhibiting the activation of CA1-ACC projecting neurons (Calcium level). (A) Timeline of CNO/saline i.p., session 1 training and Calcium signals fiber photometry recording. (B) Representative Calcium signals transient of CA1-ACC projecting neurons during session 1 training of saline-injected (B) or CNO-injected (C); vertical axis, 10% ΔF/F%, horizontal axis, 10 s; gradient of heatmap, -5%-10% (ΔF/F%). (D) Probability distribution of averaged ΔF/F% of Ca2+ signals of CA1-ACC projecting neurons between hM4Di_Saline and hM4Di_CNO groups in session 1 (*n*=4, trial= 17 each). (E) Comparison between the averaged Ca2+ signals of CA1-ACC projecting neurons between hM4Di_Saline and hM4Di_CNO groups in session 1 (*n*=4, trial=17 each). (F) Representative Calcium signals transient of ACC neurons during session 1 training of saline-injected rats (F) or CNO-injected rats (G); vertical axis, 10% ΔF/F%, horizontal axis, 10 s; gradient of heatmap, 0%-6% (ΔF/F%). (H) Probability distribution of averaged ΔF/F% of Ca2+ signals of ACC neurons between hM4Di_Saline and hM4Di_CNO groups of rats in session 1 (*n*=4, trial= 16 in hM4Di_saline group, *n*=4 trials=18 in hM4Di_CNO group). (I) Comparison between the averaged Ca2+ signals of ACC neurons between hM4Di_Saline and hM4Di_CNO groups of rats in session 1 (*n*=4, trial= 16 in hM4Di_saline group, *n*=4 trials=18 in hM4Di_CNO group). (J) Left, schematic of CA1-NAc projecting neurons experiment: AAV2-retro-CaMKII-Cre was injected into the NAc, and AAV2/9-ef1α-DIO-EYFP together with AAV8-GFAP-hM4Di–mCherry were injected into the CA1; scale bar, 20 µm; Right, EYFP-positive axons of CA1 projection neurons in the NAc; scale bar, 100 µm. (K) Representative images of hM4Di in astrocytes (red), EYFP in NAc projecting CA1 neurons (green) and c-Fos (magenta) in the CA1 of saline-injected rats (K) or CNO-injected rats (L). (M) Representative images of c-Fos (red) in NAc of saline-injected rats (M) or CNO-injected (N). (O) Comparison of the percent of CA1 cells projecting into the NAc that express c-Fos between session 1 (*n*=5) and home-cage group (*n*=4). (P) Comparison of c-Fos expression level in NAc between session 1 (*n*=5) and home-cage group (*n*=4). (Q) Comparison of the percent of CA1 cells projecting into the NAc that express c-Fos between hM4Di_CNO and hM4Di_Saline groups (*n*=5 in hM4Di_Saline, *n*=4 in hM4Di_CNO group). (R) Comparison of c-Fos expression level in NAc between hM4Di_CNO (*n*=4) and hM4Di_Saline group (*n*=5). Probability distribution are presented as the mean ± SD, other data are presented as the mean ± SEM, *p<0.05, ***p<0.001, ns *p*>0.05.

**Additional file 4:** **Fig S4.** CA1 astrocyte Gi activation interrupts middle stage of memory schema development by impairing CA1-ACC neurons’ interaction (Calcium level). (A) Timeline of CNO/saline i.p., session 10 training and Calcium signals fiber photometry recording. (B) Representative Calcium signals transient of CA1-ACC projecting neurons during session 10 training of saline-injected (B) or CNO-injected (C); vertical axis, 10% ΔF/F%, horizontal axis, 10 s; gradient of heatmap, -5%-10% (ΔF/F%). (D) probability distribution of averaged ΔF/F% of Ca2+ signals of CA1-ACC projecting neurons between hM4Di_Saline and hM4Di_CNO groups in session 10 (*n*=5, trial= 21 each). (E) comparison between the averaged Ca2+ signals of CA1-ACC projecting neurons between hM4Di_Saline and hM4Di_CNO groups in session 10 (*n*=5, trial=21 each). (F) Representative Calcium signals transient of ACC neurons during session 10 training of saline-injected rats (F) or CNO-injected rats (G); vertical axis, 10% ΔF/F%, horizontal axis, 10 s; gradient of heatmap, 0%-6% (ΔF/F%). (H) Probability distribution of averaged ΔF/F% of Ca2+ signals of ACC neurons between hM4Di_Saline and hM4Di_CNO groups of rats in session 10 (*n*=4, trial= 16 in hM4Di_saline group, *n*=4 trials=19 in hM4Di_CNO group). (I) Comparison between the averaged Ca2+ signals of ACC neurons between hM4Di_Saline and hM4Di_CNO groups of rats in session 10 (*n*=4, trial= 16 in hM4Di_saline group, *n*=4 trials=19 in hM4Di_CNO group). Probability distribution are presented as the mean ± SD, other data are presented as the mean ± SEM, ***p<0.001.

**Additional file 5:** **Fig S5.** CA1 astrocytic Gi-pathway activation has less impact on memory recall of OPAs. (A) Schematic of the experimental protocol; left, timeline of habituation, schema training, and memory recall of OPAs; right, timeline of CNO/saline i.p. before PT4-7. (B) Performance index of rats during the learning of OPA in hM4Di_CNO and hM4Di_Saline groups (*n*=7 in hM4Di_CNO group and *n*=6 in hM4Di_Saline group). CNO or saline was intraperitoneally injected in PT4-7, the memory retrieval probe test for OPA. (C) Nonrewarded cued-recall probe tests (PT1–3) for the acquisition of OPAs across sessions 2, 9, and 17 in hM4Di_CNO and hM4Di_Saline groups (*n*=7 in hM4Di_CNO group and *n*=6 in hM4Di_Saline group). The graph represents the percentage of dig time at the cued location (light color bars) relative to that of the non-cued locations (dark color bars). (D) Memory recall in PT4-7 for OPAs after PAs training in hM4Di_CNO and hM4Di_Saline groups (*n*=7 in hM4Di_CNO group and *n*=6 in hM4Di_Saline group). The graph represents the percentage of dig time at the cued location (light color bars) relative to that of the non-cued locations (dark color bars). Data are presented as the mean ± SEM, ns *p*>0.05.

**Additional file 6.**

## Data Availability

All data generated or analyzed during this study are included in this published article, its supplementary information files and publicly available repositories. Individual data values are provided in Additional File 6.

## Abbreviations

HPC: Hippocampus
ACC: Anterior cingulate cortex
PFC: Prefrontal cortex
DREADDs: Designer receptors exclusively activated by designer drugs
PAs: Paired associates
OPAs: Original paired associates
NPAs: New paired associates
CNO: Clozapine N-oxide
DMSO: Dimethyl sulfoxide
PT: Probe test
dig time %: Digging time percentage
PI: Performance index
PFA: Paraformaldehyde
NAc: Nucleus accumbens
