# Supplementary figures and images for: Astrocytes in CA1 modulate schema establishment in the hippocampal-cortical neuron network
Source: BMC Biol. 2022 Nov 10;20:250. doi: 10.1186/s12915-022-01445-6 (PMC9648012; doi:10.1186/s12915-022-01445-6)

A

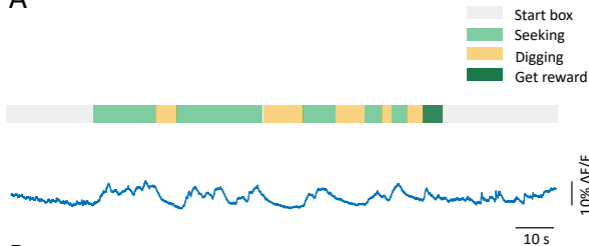

B

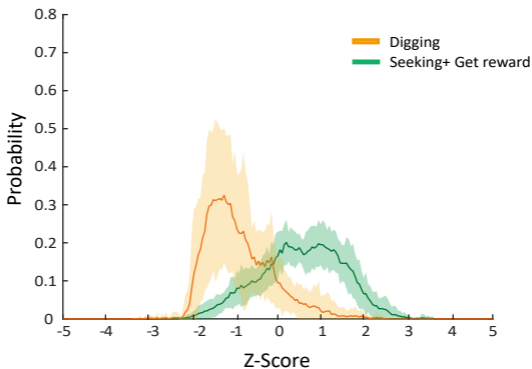

Supplement: Supplementary file 1 — Additional file 1: Fig S1. Calcium signals distribution during the trials of PA training. (A) Representative Ca2+ transient during one trial in PA training; Ca2+ signals’ peak present to seeking and get rewarding behaviors, and be flatten at digging behavior. (B) Probability distribution of averaged Z-score of Ca2+ signals in seeking + get rewarding behaviors and digging behavior for normal rats in session 1 (n=4, trials=16). [file 12915_2022_1445_MOESM1_ESM.pdf]

A

Saline/Home-cage

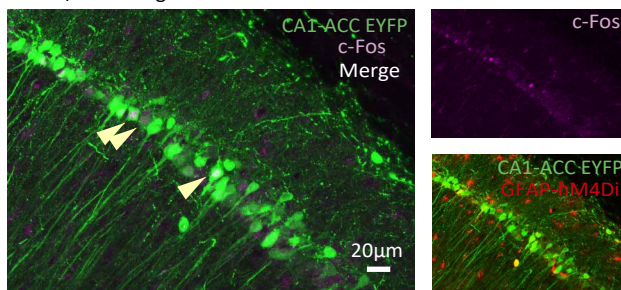

B

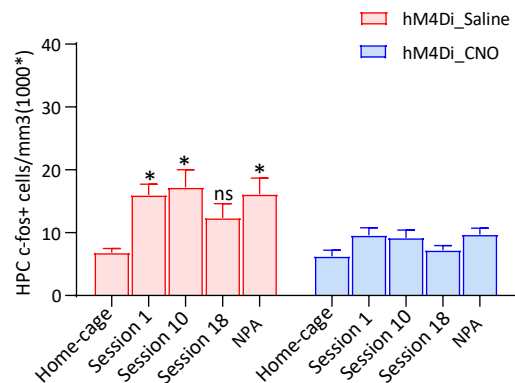

C

CNO/Home-cage

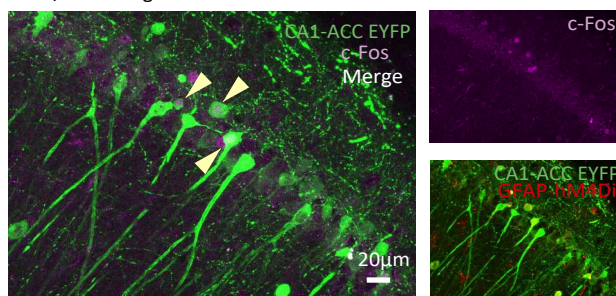

D

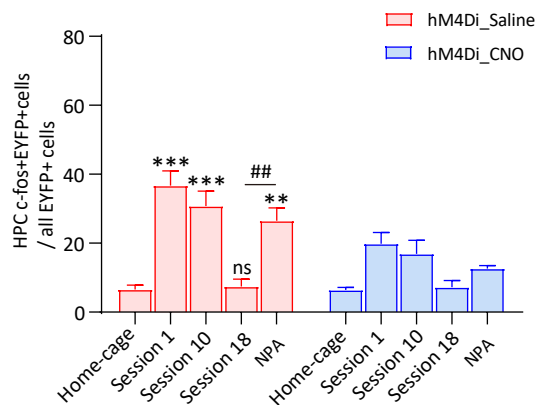

E

Saline/Home-cage

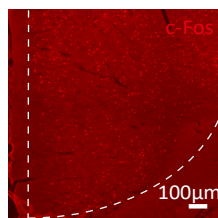

CNO/Home-cage

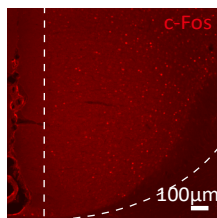

F

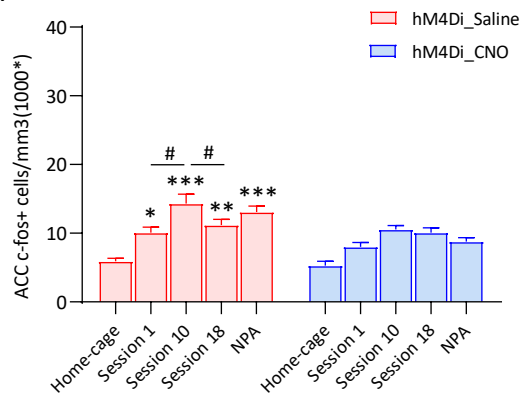

Supplement: Supplementary file 2 — Additional file 2: Fig S2. Comparison of c-Fos expression between home-cage and other sessions. (A) Representative images of hM4Di in astrocytes (red), EYFP in ACC projecting CA1 neurons (green) and c-Fos (magenta) in the CA1 of Saline-injected home-cage rats (A) or CNO-injected home-cage rats (C); white neurons that arrows point to are overlap of EYFP and c-Fos; scale bar, 20 µm. (B) Comparison of c-Fos expression level in CA1 between home-cage and sessions 1, 10, 18, NPA. (D) Comparison of the percent of CA1 cells projecting into the ACC that express c-Fos between home-cage and sessions 1, 10, 18, NPA. (E, F) Comparison of c-Fos expression level in ACC between home-cage and sessions 1, 10, 18, NPA. (G) The scheme of microdialysis probe insert and perfusion in CA1 and ACC. Data are presented as the mean ± SEM, *p<0.05, **p<0.01, ***p<0.001; #p<0.05, ##p<0.01. [file 12915_2022_1445_MOESM2_ESM.pdf]

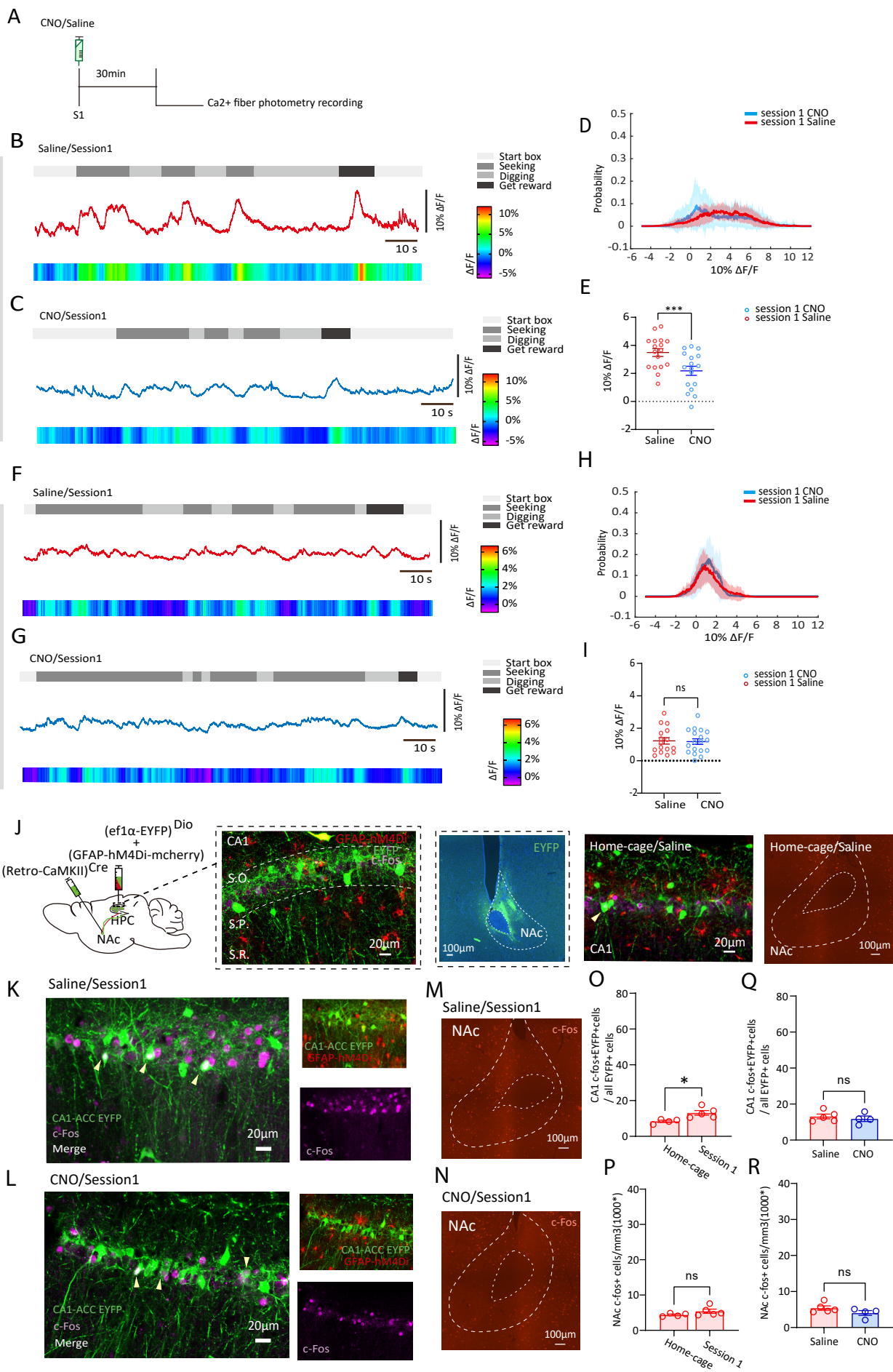

Supplement: Supplementary file 3 — Additional file 3: Fig S3. CA1 astrocyte Gi activation impairs initial schema memory by inhibiting the activation of CA1-ACC projecting neurons (Calcium level). (A) Timeline of CNO/saline i.p., session 1 training and Calcium signals fiber photometry recording. (B) Representative Calcium signals transient of CA1-ACC projecting neurons during session 1 training of saline-injected (B) or CNO-injected (C); vertical axis, 10% ΔF/F%, horizontal axis, 10 s; gradient of heatmap, -5%-10% (ΔF/F%). (D) Probability distribution of averaged ΔF/F% of Ca2+ signals of CA1-ACC projecting neurons between hM4Di_Saline and hM4Di_CNO groups in session 1 (n=4, trial= 17 each). (E) Comparison between the averaged Ca2+ signals of CA1-ACC projecting neurons between hM4Di_Saline and hM4Di_CNO groups in session 1 (n=4, trial=17 each). (F) Representative Calcium signals transient of ACC neurons during session 1 training of saline-injected rats (F) or CNO-injected rats (G); vertical axis, 10% ΔF/F%, horizontal axis, 10 s; gradient of heatmap, 0%-6% (ΔF/F%). (H) Probability distribution of averaged ΔF/F% of Ca2+ signals of ACC neurons between hM4Di_Saline and hM4Di_CNO groups of rats in session 1 (n=4, trial= 16 in hM4Di_saline group, n=4 trials=18 in hM4Di_CNO group). (I) Comparison between the averaged Ca2+ signals of ACC neurons between hM4Di_Saline and hM4Di_CNO groups of rats in session 1 (n=4, trial= 16 in hM4Di_saline group, n=4 trials=18 in hM4Di_CNO group). (J) Left, schematic of CA1-NAc projecting neurons experiment: AAV2-retro-CaMKII-Cre was injected into the NAc, and AAV2/9-ef1α-DIO-EYFP together with AAV8-GFAP-hM4Di–mCherry were injected into the CA1; scale bar, 20 µm; Right, EYFP-positive axons of CA1 projection neurons in the NAc; scale bar, 100 µm. (K) Representative images of hM4Di in astrocytes (red), EYFP in NAc projecting CA1 neurons (green) and c-Fos (magenta) in the CA1 of saline-injected rats (K) or CNO-injected rats (L). (M) Representative images of c-Fos (red) in NAc of [file 12915_2022_1445_MOESM3_ESM.pdf]

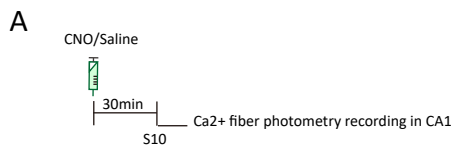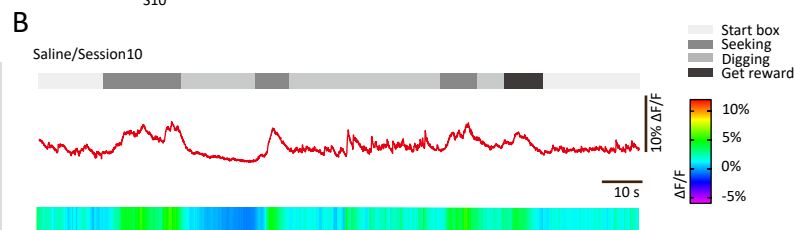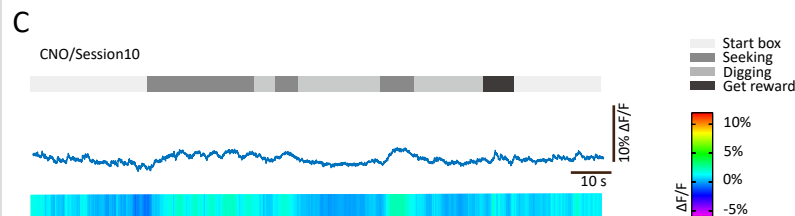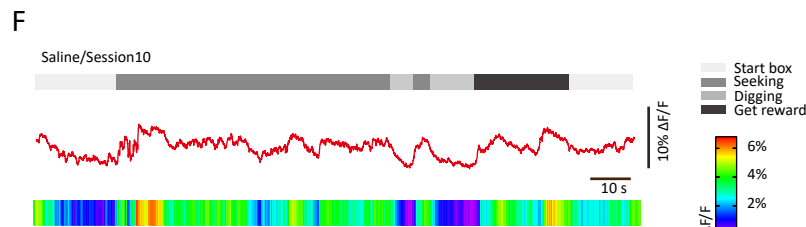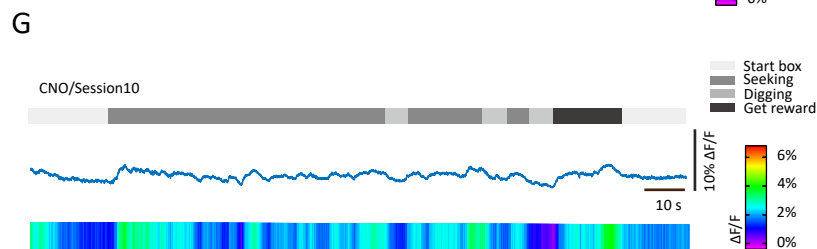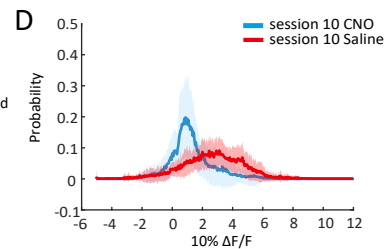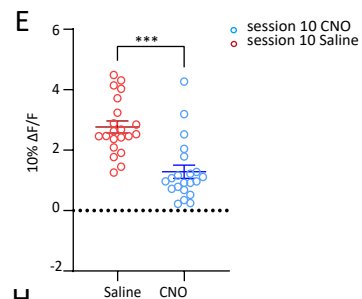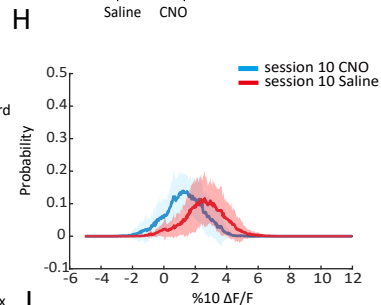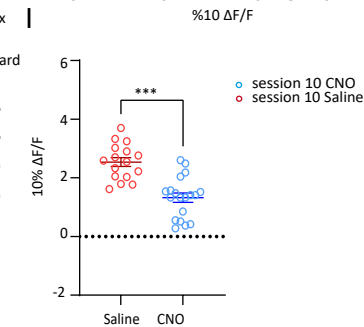

HPC/CA1

ACC

Supplement: Supplementary file 4 — Additional file 4: Fig S4. CA1 astrocyte Gi activation interrupts middle stage of memory schema development by impairing CA1-ACC neurons’ interaction (Calcium level). (A) Timeline of CNO/saline i.p., session 10 training and Calcium signals fiber photometry recording. (B) Representative Calcium signals transient of CA1-ACC projecting neurons during session 10 training of saline-injected (B) or CNO-injected (C); vertical axis, 10% ΔF/F%, horizontal axis, 10 s; gradient of heatmap, -5%-10% (ΔF/F%). (D) probability distribution of averaged ΔF/F% of Ca2+ signals of CA1-ACC projecting neurons between hM4Di_Saline and hM4Di_CNO groups in session 10 (n=5, trial= 21 each). (E) comparison between the averaged Ca2+ signals of CA1-ACC projecting neurons between hM4Di_Saline and hM4Di_CNO groups in session 10 (n=5, trial=21 each). (F) Representative Calcium signals transient of ACC neurons during session 10 training of saline-injected rats (F) or CNO-injected rats (G); vertical axis, 10% ΔF/F%, horizontal axis, 10 s; gradient of heatmap, 0%-6% (ΔF/F%). (H) Probability distribution of averaged ΔF/F% of Ca2+ signals of ACC neurons between hM4Di_Saline and hM4Di_CNO groups of rats in session 10 (n=4, trial= 16 in hM4Di_saline group, n=4 trials=19 in hM4Di_CNO group). (I) Comparison between the averaged Ca2+ signals of ACC neurons between hM4Di_Saline and hM4Di_CNO groups of rats in session 10 (n=4, trial= 16 in hM4Di_saline group, n=4 trials=19 in hM4Di_CNO group). Probability distribution are presented as the mean ± SD, other data are presented as the mean ± SEM, ***p<0.001. [file 12915_2022_1445_MOESM4_ESM.pdf]

A

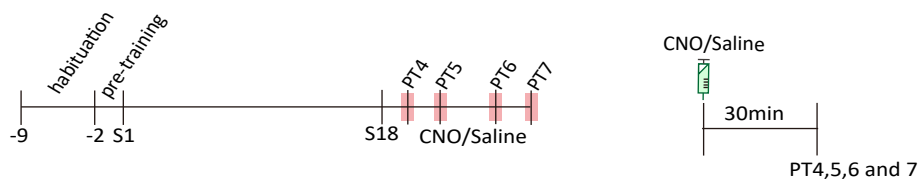

B

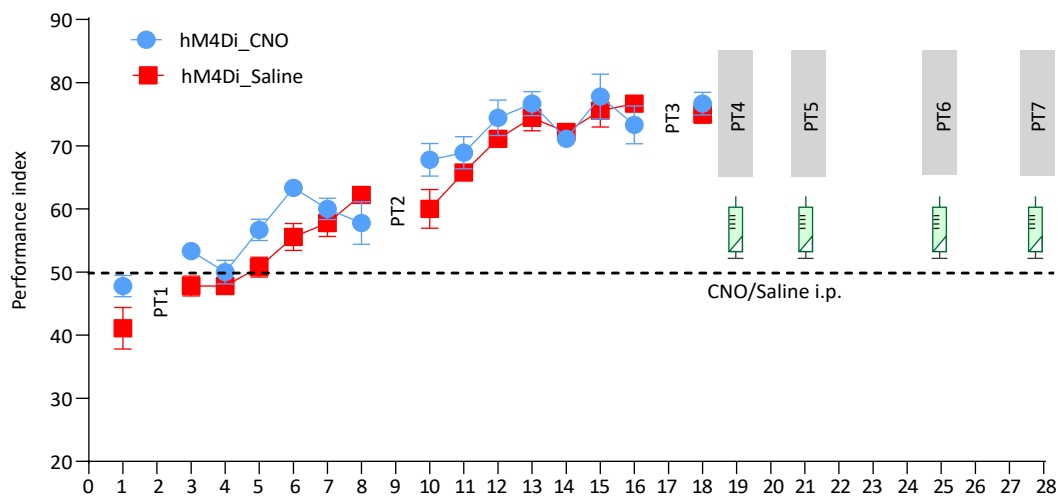

C

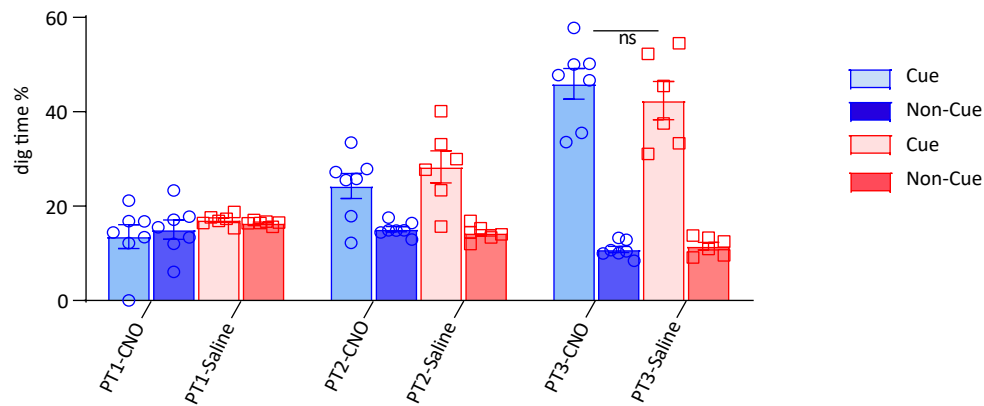

D

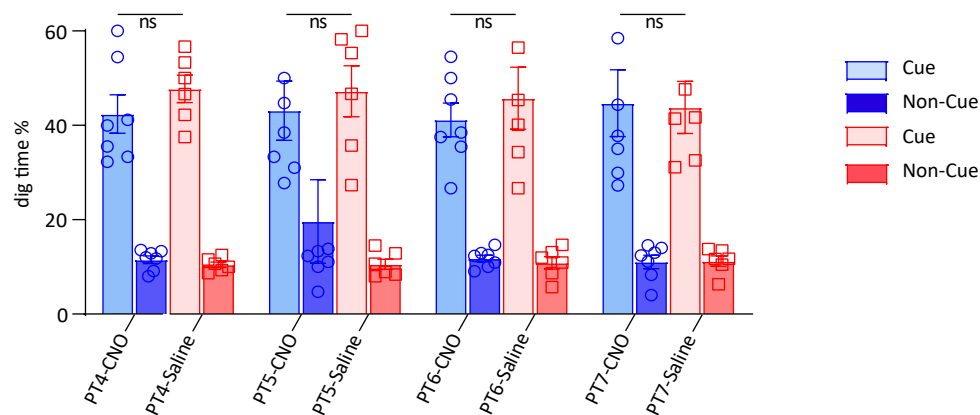

Supplement: Supplementary file 5 — Additional file 5: Fig S5. CA1 astrocytic Gi-pathway activation has less impact on memory recall of OPAs. (A) Schematic of the experimental protocol; left, timeline of habituation, schema training, and memory recall of OPAs; right, timeline of CNO/saline i.p. before PT4-7. (B) Performance index of rats during the learning of OPA in hM4Di_CNO and hM4Di_Saline groups (n=7 in hM4Di_CNO group and n=6 in hM4Di_Saline group). CNO or saline was intraperitoneally injected in PT4-7, the memory retrieval probe test for OPA. (C) Nonrewarded cued-recall probe tests (PT1–3) for the acquisition of OPAs across sessions 2, 9, and 17 in hM4Di_CNO and hM4Di_Saline groups (n=7 in hM4Di_CNO group and n=6 in hM4Di_Saline group). The graph represents the percentage of dig time at the cued location (light color bars) relative to that of the non-cued locations (dark color bars). (D) Memory recall in PT4-7 for OPAs after PAs training in hM4Di_CNO and hM4Di_Saline groups (n=7 in hM4Di_CNO group and n=6 in hM4Di_Saline group). The graph represents the percentage of dig time at the cued location (light color bars) relative to that of the non-cued locations (dark color bars). Data are presented as the mean ± SEM, ns p>0.05. [file 12915_2022_1445_MOESM5_ESM.pdf]
